# The mechanochemistry of copper reports on the directionality of unfolding in model cupredoxin proteins

**DOI:** 10.1038/ncomms8894

**Published:** 2015-08-03

**Authors:** Amy E. M. Beedle, Ainhoa Lezamiz, Guillaume Stirnemann, Sergi Garcia-Manyes

**Affiliations:** 1 Department of Physics, King’s College London, London WC2R 2LS, UK; 2 Randall Division of Cell and Molecular Biophysics, King’s College London, London WC2R 2LS, UK; 3 CNRS - Institut de Biologie Physico-Chimique - PSL Research University, Laboratoire de Biochimie Théorique, 75005 Paris, France

## Abstract

Understanding the directionality and sequence of protein unfolding is crucial to elucidate the underlying folding free energy landscape. An extra layer of complexity is added in metalloproteins, where a metal cofactor participates in the correct, functional fold of the protein. However, the precise mechanisms by which organometallic interactions are dynamically broken and reformed on (un)folding are largely unknown. Here we use single molecule force spectroscopy AFM combined with protein engineering and MD simulations to study the individual unfolding pathways of the blue-copper proteins azurin and plastocyanin. Using the nanomechanical properties of the native copper centre as a structurally embedded molecular reporter, we demonstrate that both proteins unfold via two independent, competing pathways. Our results provide experimental evidence of a novel kinetic partitioning scenario whereby the protein can stochastically unfold through two distinct main transition states placed at the N and C termini that dictate the direction in which unfolding occurs.

## Abstract (web summary)

In metalloproteins, a metal cofactor participates in the formation of the correct fold. Here the authors demonstrate—using single molecule force spectroscopy and the native copper centre as an embedded internal reporter—that the blue-copper proteins azurin and plastocyanin unfold via two independent competing pathways under force.

## Graphical abstract

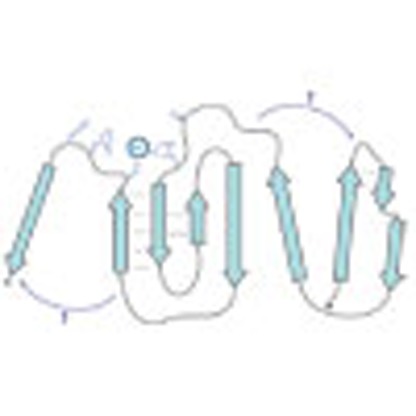

During folding, unfolded polypeptides progressively narrow down their conformational space search, often briefly visiting intermediate conformations reminiscent of localized energy minima^1^,^2^,^3^ until they eventually reach the functional natively folded structure^4^,^5^,^6^,^7^,^8^. Understanding the molecular mechanisms underpinning each intermediate as well as identifying the sequential order statistics in which each conformation occurs along an individual (un)folding pathway, remains a challenge in the protein folding field^2^.

Single molecule experiments are ideally suited to detect individual short-lived protein conformations, often occurring through statistically rare pathways that would be otherwise smeared out in the ensemble. In particular, force experiments excel at precisely mapping out individual mechanical (un)folding pathways by monitoring the protein extension along a well-defined one-dimension reaction coordinate; the protein’s end-to-end length. These experiments have revealed signatures of complexity in the unfolding trajectories of a wide variety of proteins. While earlier works reported the presence of well-defined mechanical intermediates for a number of topologically distinct small proteins^9^,^10^,^11^,^12^,^13^, more recent studies on larger multidomain proteins have experimentally demonstrated the concept of kinetic partitioning^14^,^15^,^16^,^17^, where both the number and the position of these intermediates stochastically change after the main unfolding event, which remains the same in all trajectories^18^,^19^,^20^,^21^,^22^,^23^,^24^. A further layer of complexity would be added if a protein also exhibited stochastic behaviour in the rupture of the main mechanical clamp, defining a multiplicity of pathways from the early stages of unfolding.

A key advantage of single molecule atomic force microscopy (AFM) mechanical experiments is the ability to directly measure the mechanical activation of individual chemical bonds. These measurements enable a direct correlation between the strength and nature of the bond and its mechanical stability. For example, while mechanical protein unfolding involves the disruption of a key set of hydrogen bonds, typically requiring forces ranging from ∼20 to 200 pN (ref. 25), the mechanical stability of covalent bonds is significantly higher, up to ∼3 nN (ref. 26). In particular, covalent-disulfide bonds cannot be broken on the application of force, unless a nucleophile is added to the solution^27^. The repertoire of chemical interactions that are ruptured during the mechanical unfolding trajectories have recently been extended to include organometallic bonds^22^,^28^. Metals play an important structural and biological role in a vast variety of proteins, spanning one-half of all known protein crystal structures in the Protein Data Bank. Hence, understanding the mechanochemistry of individual organometallic bonds and the kinetics of metal release from the protein core is essential when studying the mechanical (un)folding of metalloproteins. While pioneering recent studies have mainly focused on the mechanical properties of iron-binding proteins such as rubredoxin^22^,^28^,^29^, the nanomechanics of other organometallic bonds such as copper, which is an essential metal in biological processes involved in a variety of redox reactions, remains vastly elusive.

Here we use a combination of protein engineering and single molecule force experiments to study the individual unfolding pathways of two model blue-copper proteins: azurin, involved in electron-transfer processes in the redox system of *Pseudomonas aeruginosa* and other bacteria^30^, and plastocyanin^31^,^32^,^33^, which functions as an electron carrier in the photosynthetic electron-transfer chain, shuttling electrons between the cytochrome b6f complex and Photosystem I in all higher plants as well as in a number of green algae and cyanobacteria such as *Phormidium laminosum*^34^. Azurin is topologically rich, exhibiting a β-barrel structure organized in a Greek key motif, Fig. 1a,b (refs 35, 36). Crucially, its structure harbours the three main families of chemical determinants that confer mechanical stability to proteins, namely, the presence of two sets of key hydrogen bonds at each of its termini, a penta-coordinated copper ion and a native covalent-disulfide bond. Such interesting structural diversity renders azurin particularly attractive from the mechanochemistry perspective. Using the native copper as an internal ‘built-in’ mechanical intermediate reporter, we demonstrate that azurin can unfold via two competing independent pathways. We show that the protein can stochastically unfold along either the N–C or the C–N direction, suggesting that the barriers to unfolding from both termini are largely equivalent at the probed pulling velocity. Each individual unfolding pathway is unambiguously distinguished by the position of the subsequent rupture of the copper-ligand bond and further supported by directed mutagenesis and molecular dynamics (MD) simulations. Crucially, the mechanical stability of each individual organocopper bond is surprisingly low despite of their highly covalent character. These observations are expanded to the topologically similar plastocyanin protein, exhibiting analogous behaviour. Our results provide experimental evidence of a novel kinetic partitioning scenario, where the stochastic nature of the unfolding process originates already at the very early stages, as the protein chooses between two competing main unfolding transition states that precisely dictate the unravelling sense along the termini direction.

## Results

### The nanomechanics of azurin unfolding

**Figure 1:**
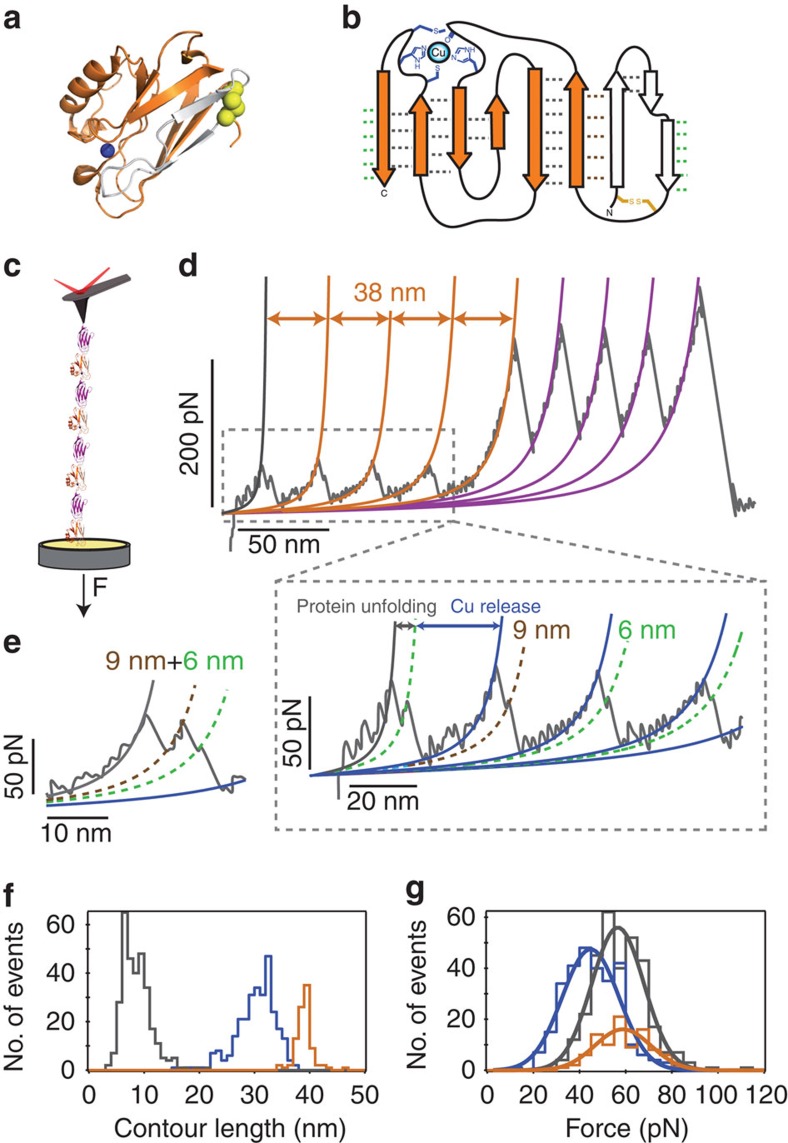
Wt-azurin unfolds through two mechanically labile intermediates. (**a**) Schematic structure of wt-azurin (PDB: 4AZU). (**b**) The residues trapped after the disulfide bond (yellow) are marked in white. The rest are represented in orange. (**c**) Scheme of the engineered (Azu–I27)_4_ polyprotein being pulled in the AFM set-up. (**d**) Pulling on an individual (Azu-I27)_4_ polyprotein results in an unfolding trajectory, where the unfolding of azurin (orange) occurs at ∼55 pN, followed by the unfolding of the I27 domains, distinguished by an unfolding force of ∼210 pN and an increment in contour length of Δ*L*=28 nm (purple). *Inset*: The unfolding of wt-azurin occurs via two well-defined mechanically stable intermediates occurring at ∼6 nm (green dashed line) or ∼9 nm (brown dashed line). (**e**) In a few trajectories (*n*=25), a four-state unfolding pathway is measured, where both intermediates (∼6 and ∼9 nm) are captured within the same trajectory. (**f**,**g**) Worm-like chain fits to the data reveal that each complete azurin unfolding event occurs concomitant to an increment in contour length of Δ*L*=38.6±0.9 nm, *n*=93 (**f**, orange histogram), requiring an unfolding force of 56.3±12.1 pN, *n*=93 (**g**, orange). When unfolding occurs via a mechanical intermediate, the main unfolding event (dashed lines), occurring at 54.1±11.3 pN, *n*=323 (**g**, grey), elicits a protein length of <10 nm (**f**, grey histogram) before it reaches a mechanically resistant intermediate. The mechanical disruption of such intermediate form occurs at forces of ∼42.3±12.3 pN, *n*=289 (**g**, blue), with a concomitant protein elongation of Δ*L*∼30 nm (**f**, blue histogram).

Using single molecule spectroscopy AFM we stretched a single azurin polyprotein whereby each azurin monomer is flanked by two modules of the titin 27th Ig domain, which provide an internal molecular fingerprint^37^ (Fig. 1c). A typical force-extension unfolding trajectory of the (Azu–I27)_4_ polyprotein marks first the unfolding of the azurin proteins (orange), occurring at ∼56 pN, followed by the unfolding of the I27 modules (purple), requiring ∼210 pN to unfold (Fig. 1d) and exhibiting an increase in contour length of Δ*L* ∼28 nm (ref. 37). Fitting the worm-like chain model of polymer elasticity (WLC) to the data reveals that each azurin unfolding event entails a concomitant increase in contour length of Δ*L* ∼38 nm (orange fits). Assuming a contour length per residue of 0.38 nm (ref. 38), unfolding and extending each azurin monomer right from the termini would release Δ*L*∼48 nm. However, a disulfide bond between the amino acids C3–C26 shortcuts the protein (Fig. 1a,b, white), and effectively shortens it by ∼10 nm. Hence, the increase of Δ*L* ∼38 nm that we observed is perfectly compatible with the unfolding of the whole azurin protein from a position very close to the termini in the presence of the disulfide bond (orange region in Fig. 1a, Supplementary Fig. 1 and Supplementary Table 1).

Close inspection of each azurin unfolding event (Fig. 1d, inset) reveals, in ∼80% of the occurrences, the presence of a clear mechanical intermediate (Supplementary Fig. 2). Remarkably, the position of this intermediate is not unique, since we measured (with similar probability) events occurring either ∼6 nm (dashed green fit) or ∼9 nm (dashed brown fit, Fig. 1d, inset) after the main unfolding event, occurring at ∼54 pN (Fig. 1g, grey). Despite the close proximity in the measured length defining both intermediates, the grey histogram in Fig. 1f is bimodal and the presence of both intermediates is statistically significant, with >99.9% certainty (Hartigan’s diptest, Supplementary Fig. 3). The mechanical disruption of such intermediate conformations occurs at forces of ∼43 pN (Fig. 1g, blue), with a concomitant protein elongation of Δ*L*∼32 or Δ*L*∼28 nm, respectively (Fig. 1f, blue histogram). Remarkably, in 8% (*n*=25) of the total unfolding trajectories showing an intermediate, the protein unfolds via a four-state unfolding pathway, where both intermediates (∼6 and ∼9 nm) are readily captured (Fig. 1e). Crucially, in these cases the order of occurrence of both ∼6 and ∼9 nm events is stochastic. In the remaining ∼20% of events, the protein unfolds through a simple two-state scenario, extending a total length of ∼38 nm, requiring forces of ∼56 pN (Fig. 1g, orange). To further validate the presence of the two distinct mechanical intermediates, we conducted experiments under force-clamp conditions, whereby the protein extension is recorded as a function of time (Supplementary Fig. 4). These experiments confirmed the presence of two statistically distinct mechanical intermediates. Details on the detection of unfolding intermediates in constant velocity and force-clamp experiments are shown in Supplementary Figs 5 and 6, respectively.

According to the presence of the copper site in the tertiary structure of azurin, we speculate that the mechanical intermediates that we measured are associated with the mechanical rupture of the copper centre. In azurin (PDB: 4AZU), copper exhibits a characteristic trigonal bipyramidal geometry, whereby the metal interacts with five key resides; Met-121, His-117, Cys-112, His-46 and Gly-45 (Fig. 1b). Of these, structural and spectroscopic studies reveal that the three equatorial residues Cys-112, His-117 and His-46 exhibit the strongest interactions with the metal^31^. Taking this into consideration, the ∼6-nm intermediate would be compatible with an unfolding scenario where the protein unfolds from the C terminus starting by disrupting the β-sheets β2b–β8 (Fig. 1b, green hydrogen bonds) up to the Cu–S_Cys112_ bond, hence releasing the amino acids contained between the C terminus (Lys-128) and Cys-112 (Supplementary Fig. 1 and Supplementary Table 1). However, the ∼9nm intermediate event cannot be accounted for by this unfolding sequence. By contrast, it could be readily explained assuming that the protein unfolds from the N terminus instead (Fig. 1b, brown hydrogen bonds). In that case, the position of the mechanical intermediate would match perfectly with the unfolding up to the Cu–N_His46_ bond. Indeed, extending the amino acids released on unfolding the protein from the N terminus up to His-46 (after deducting those residues that are trapped behind the disulfide bond formed between Cys-3 and Cys-26, white region in Fig. 1a,b) results in a length increment of ∼11 nm (Supplementary Fig.1 and Supplementary Table 1), consistent with the observed ∼9nm intermediate. Due to the weak character of the backbone carbonyl of Gly-45 (ref. 39), it is highly unlikely that the rupture of such interaction with the Cu centre can account for the mechanical intermediate observed in our experiments.

### The distinct unfolding pathways revealed by mutagenesis

**Figure 2:**
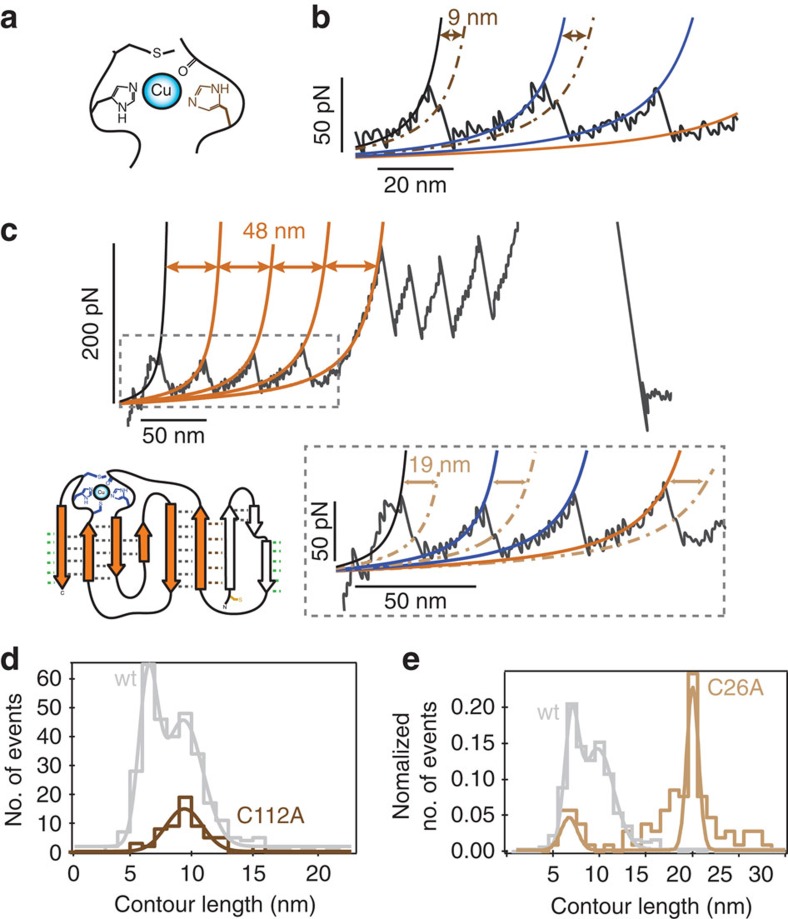
The Azu_C112A_ and the Azu_C26A_ mutations confirm the existence of parallel unfolding pathways. (**a**) The Azu_C112A_ mutation prevents the formation of the Cu–S_Cys112_ bond. (**b**) On unfolding, the Azu_C112A_ protein extends by ∼9 nm (dashed brown fit) before encountering a mechanical intermediate that corresponds to the disruption of the Cu–N_His46_ bond (dark blue fit). (**c**) The C26A mutation prevents the formation of the disulfide bond. In the absence of the disulfide bond, each azurin unfolding event occurs concomitant to an increase in contour length of Δ*L=*47.6±1.2 nm, *n*=77 (orange fits). The mechanical intermediate is now mostly observed (80% of the occurrences) at Δ*L=*19.0±0.5 nm (dashed light brown fits), *n*=78, consistent with the unfolding and extension of the total protein length from the N terminus up to His-46. In the remaining 20% of the cases in which the protein unfolds via a three-state pattern, the protein unfolds via the C terminus, as fingerprinted by the intermediate occurring at Δ*L=*5.6±1.1 nm, *n*=18. (**d**) The histogram corresponding to the increment in contour length elicited after the main unfolding event measured for the Azu_C112A_ mutant reveals a main unfolding event occurring at Δ*L=*9.4±1.5 nm, *n*=62 nm, which matches rather well the broader histogram of the wt-Azu data shown in Fig. 1f (background light grey bars), containing both intermediate events. (**e**) The normalized histogram corresponding to the unfolding of the Azu_C26A_ mutant shows well-defined intermediates, occurring at Δ*L=*6.2±1.0 nm, *n*=18 and Δ*L=*19.5±0.7 nm, *n*=81. Crucially, the N terminus pathway is highly favoured in the absence of the native disulfide bond.

To verify the hypothesis that azurin unfolds via two competing pathways fingerprinted by the rupture of the Cu–S and Cu–N individual bonds, we first engineered a polyprotein containing the Cys112Ala mutant^40^, thus removing the Cu–S_Cys112_ bond (Fig. 2a). Crucially, circular dichroism experiments certified the correct folding of the protein (Supplementary Fig. 7) and inductively coupled plasma–optical emission spectroscopy (ICP-OES) measurements revealed that, although only partially, Cu was still embedded within the azurin structure (Supplementary notes). Stretching the (Azu_C112A_–I27)_4_ polyprotein revealed the presence of only the ∼9nm intermediate (brown fits, Fig. 2b; and brown histogram, Fig. 2d). The absence of the ∼6nm intermediate in the Azu_C112A_ case firmly suggests that the ∼6nm intermediate observed in the unfolding trajectories of wildtype (wt)-Azu (Fig. 1d) is indeed triggered by the mechanical disruption of the Cu–S_Cys112_ bond.

To further investigate the origin of the ∼9nm intermediate, we studied the unfolding properties of the (Azu_C26A_–I27)_4_ polyprotein, lacking the native disulfide bond (Fig. 2c). The absence of the disulfide bond was further checked by the Ellman’s assay^41^,^42^, revealing an increase in the number of free sulfhydryls in the polyprotein chain in the mutated protein as opposed to the wt-form (Supplementary notes). As expected, each azurin unfolding event showed a larger associated increment in contour length of Δ*L* ∼48 nm, corresponding to the full protein length (orange fits). Akin to the wt- case, the unfolding trajectories showed two well-defined intermediates; the first one occurring at ∼6 nm and the second one now occurring at ∼19 nm (dashed light brown fits). The distribution of events is shown in the contour length histogram of Fig. 2e (light brown). Hence, on removal of the disulfide bond, only the N terminus unfolding pathway was modified by adding to the length required to reach the His-46 the protein length previously trapped behind the disulfide bond resulting in the total length increase of ∼19 nm, thus separating both populations of intermediate events. Interestingly, the relative abundance of both pathways for this disulfide bond-depleted construct is not ∼50% as for the wt- construct; rather, the ‘N-terminus’ pathway, eliciting the 19-nm intermediate, is observed in ∼80% of the events featuring an intermediate, as observed in the normalized histogram shown in Fig. 2e.

### The plastocyanin protein exhibits a similar unfolding scheme

**Figure 3:**
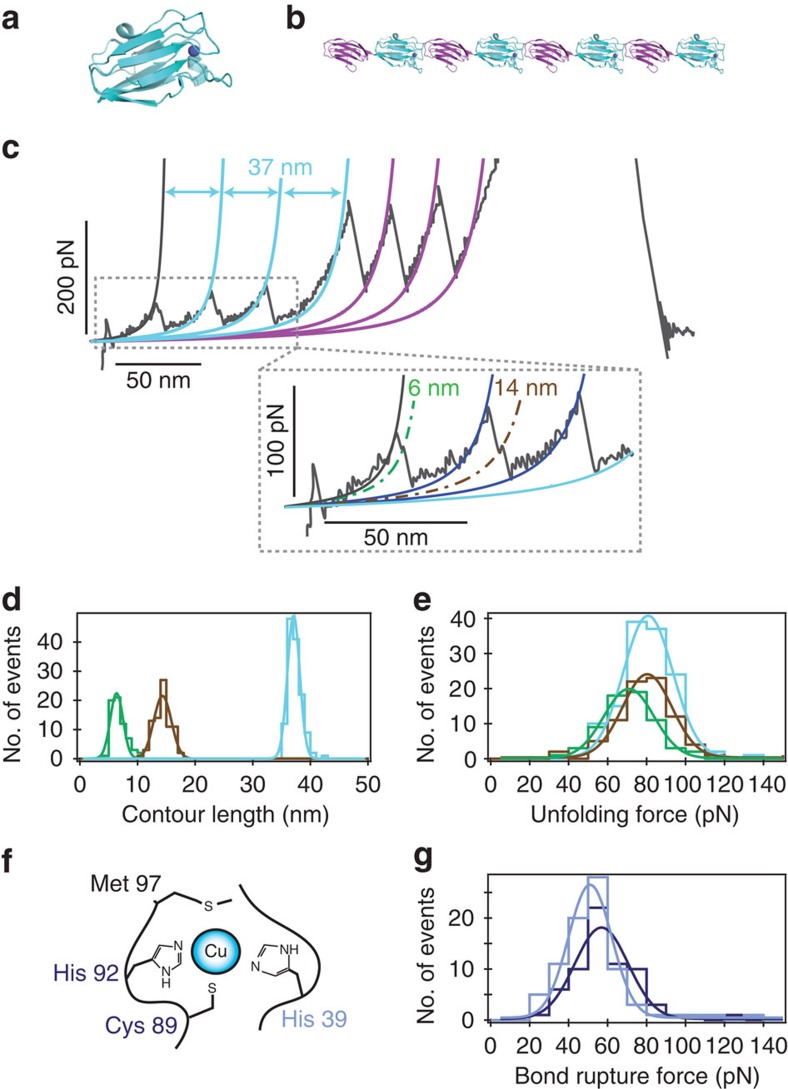
Plastocyanin unfolds through parallel pathways fingerprinted by the rupture of individual Cu–S and Cu–N bonds. (**a**) Plastocyanin structure (PDB: 3BQV). (**b**) Scheme of the (Plastocyanin-I27)_4_ polyprotein. (**c**) Individual constant velocity unfolding trajectory of a single (Plastocyanin-I27)_4_ polyprotein, whereby the unfolding of each plastocyanin domain (cyan worm-like chain (WLC) fits) occurs before the unfolding of the I27 markers (purple WLC fits). *Inset*: Close inspection to each plastocyanin unfolding event reveals that the unfolding process occurs through two distinct mechanical intermediates, which are characterized (**d**) by an increase in contour length of either Δ*L*=5.9±1.1 nm, *n*=66 (green) or Δ*L*=13.8±1.5 nm, *n*=82 (brown). In the cases where no intermediate is observed, suggestive of an apoplastocyanin form, unfolding triggers the extension of the complete length of the protein, Δ*L*=36.8±1.1 nm, *n*=135 (cyan). (**e**) Remarkably, the unfolding forces measured for each individual pathway (N terminus, C terminus and two-state unfolding) are very similar (75.3±12 pN, *n*=82, brown; 65.9±13 pN, *n*=66, green and 75.8±12 pN, *n*=135, cyan), respectively. In all cases, protein unfolding occurs at higher forces ∼76 pN than the rupture of (**f**) the Cu–N_His39_ bond (46.0±11 pN, *n*=82, light blue) or the Cu–S_Cys89_ individual bond, occurring at 51.8±13 pN, *n*=66 (dark blue), as shown in (**g**).

Azurin is a model protein for Type 1 (T1) copper centres. T1 copper proteins are distinct from other copper proteins because of their unique geometry and ligand sets^30^. Despite the fact that the sequence identity between the T1 copper proteins is <20% (ref. 43), the overall structural fold of different T1 copper proteins is highly conserved^31^. Such common cupredoxin fold consists of eight β-strands arranged into a Greek β-barrel (Supplementary Fig. 8). Inspired by such large structural homology, we investigated whether the intriguing features observed in the mechanical unfolding pathway of azurin could be generalized to other structurally similar T1 model blue-copper proteins with the cupredoxin fold, such as plastocyanin (Fig. 3a). To this end, we studied the mechanical unfolding pathways of the (Plastocyanin-I27)_4_ polyprotein (Fig. 3b). Complete unfolding of plastocyanin, occurring at ∼75 pN (Fig. 3e), results in an increase of contour length of Δ*L* ∼37 nm (Fig. 3c, cyan fits and Supplementary Fig. 9), which is consistent with the extension of the full protein (Supplementary Table 2). Remarkably, in 60% of the unfolding events a mechanical intermediate was also observed (Fig. 3c, inset), which, as in the case of azurin, clustered in two main length populations at ∼6 and ∼14 nm (Supplementary Figs. 10–12 and Supplementary Table 2), giving rise to the—now well-separated—bimodal histogram shown in Fig. 3d. Akin to azurin, in plastocyanin the copper ion is bound to the sulfur of Cys-89 and to two nitrogens (from His-39 and His-92) in the trigonal plane, and to a methionyl sulfur (from Met-97) in the axial position at a relatively longer distance (Fig. 3f). Taking this structure into account, the ∼6-nm intermediate can be readily explained in this case by the unfolding of the protein from the C terminus until Cys-89. In this case, after unfolding, the protein would be expected to extend 7.0 nm (Supplementary Fig. 9 and Supplementary Table 2). By contrast, unfolding the protein from the N terminus up to His-39 would extend 16.2 nm, thus consistent with the second intermediate that we observed. Crucially, mechanical disruption of the copper organometallic bonds requires forces of ∼45 pN (Fig. 3g).

### Molecular dynamics simulations

**Figure 4:**
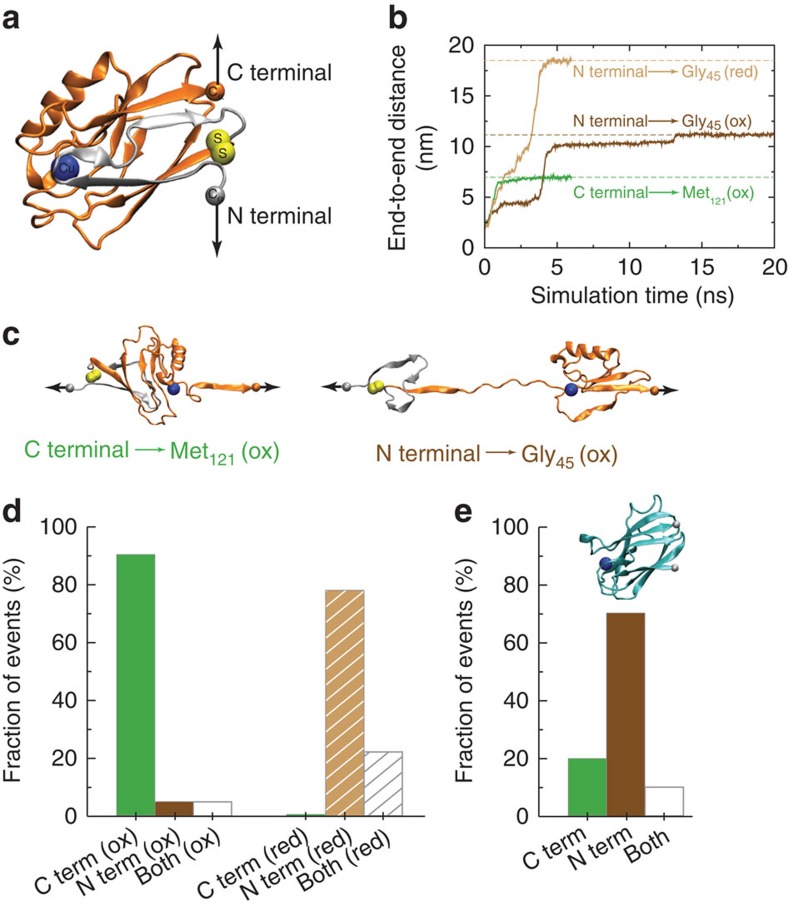
MD simulations capture the distinct unfolding pathways. (**a**) Azurin PDB structure (4AZU). Copper is explicitly shown as a blue sphere, the two sulfur atoms of the disulfide bond Cys3—Cys26 in yellow, the residues trapped after the disulfide bond in white and the rest, in orange. In a steered MD set-up, one of the termini is fixed in the laboratory frame and a constant force is applied to the other one. (**b**) Unfolding trajectories represented by the time evolution of the protein’s end-to-end distance for three representative scenarios: wt-Azu unfolding from the N-terminal up to Gly-45 (brown), from the C-terminal up to Met-121 (green) and unfolding of wt-Azu lacking its disulfide bond from the N-terminal side (light brown). Each simulation was stopped once the end-to-end distance had reached the maximum possible extension at this force, the organometallic bonds being intact. (**c**) Representative structures following unfolding on the C-terminal side and up to Met-121 (left) and unfolding on the N-terminal side up to Gly-45 (right). (**d**) Percentage of the population of events falling in each scenario (green: C-terminal unfolding; brown: N-terminal; grey: both simultaneously), for both the oxidized form of wt-Azu (solid bars) and the reduced version lacking its disulfide bond (patterned bars). (**e**) Unfolding trajectories of wt-plastocyanin (3BQV). In ∼70% of the trajectories (*n*=7), plastocyanin unfolds through the N terminus (brown). In 20% of the occurrences (*n*=2), the protein unfolds via the C terminus instead (green), and in the remaining trajectory the protein unfolds from both termini simultaneously (grey). Hence, from a qualitative viewpoint, these *in-silico* results support the experimental observations.

To gain further molecular insight into such enticing unfolding scheme, we conducted MD simulations of azurin and plastocyanin unfolding under a constant force^38^. While present force fields are not yet adequate to reliably measure the rupture of organometallic bonds, which are constrained with harmonic potentials during the course of the simulation, the simulations provide a detailed molecular picture of the mechanisms underlying the first stages of protein unfolding, where a set of key hydrogen bonds are disrupted by the stretching force. Importantly, constriction of the organocopper bonds does not significantly influence the initial stages of protein unfolding (Supplementary Fig. 13). Individual azurin unfolding trajectories were generated by pulling on one terminal C_α_ (the other being fixed) under a constant force in the range of 250–350 pN (Fig. 4a) that guarantees that unfolding occurs on a timescale accessible to the simulation (∼10 ns) as shown in Fig. 4b. In 90% of the cases (*n*=19), the protein unfolded from the C terminus while in a single trajectory (*n*=1), the protein was found to unfold from the N terminus instead (Fig. 4c and solid bars in Fig. 4d). In another individual trajectory the protein was observed to unfold from both ends simultaneously. When unfolding from the C terminus, the β2b–β8 strands unzip first, followed by the disruption of β7-β8. On the contrary, unfolding from the N terminus encompasses the rupture of the hydrogen bonds between three pairs of β-sheets, namely, β2b–β8, β1–β3 and β3–β6. In sharp contrast with these results, pulling on the reduced protein (containing free cysteines, without the disulfide bond present) revealed unfolding trajectories whereby the protein mainly unfolded (80%, *n*=7) from the N terminus (patterned bars in Fig. 4d), in close agreement with the experimental results (Fig. 2e).

Similarly, pulling on the wt-plastocyanin protein under a constant force of 250–350 pN revealed that, in 70% of the trajectories (*n*=7), the protein unfolded from the N terminus, while in 20% of the trajectories (*n*=2), the unfolding started at the C terminus instead (Fig. 4e). In an individual trajectory (*n*=1), unfolding from both termini occurred concomitantly. Very similar ratios were observed for the case of apoplastocyanin (80/20/0%, respectively, *n*=10), further validating the followed approach of constraining the organocopper bonds to study the initial stages of mechanical unfolding (Supplementary Fig. 13). Altogether, the *in-silico* unfolding trajectories demonstrate the unusual capability of both azurin and plastocyanin to mechanically unfold from both termini, lending strong support to the experimental data. Precise information of each individual simulation run is detailed in the Supplementary Tables 3–6.

## Discussion

Here we employed the mechanochemistry of copper to elucidate, at the single molecule level, the distinct mechanical unfolding pathways of the model T1 copper proteins azurin and plastocyanin. The active site of azurin contains a copper centre in a trigonally distorted tetrahedral geometry with a short Cu–S_Cys112_ bond (∼2.1 Å) and two typical Cu–N_His_ bonds (∼1.95 Å) in the equatorial plane^39^. The axial position is occupied by a methionine at a relatively longer distance Cu–S_Met121_ (2.9 Å) (ref. 31). Finally, a weak-ionic interaction from an axial carbonyl oxygen (Gly-45) is also present. Similarly, in plastocyanin Cu(II) is coordinated by four atoms, the N^δ^ atoms of His-39 (∼2.2 Å) and His-92 (∼2.0 Å), the S^γ^ atom of Cys-89 (∼2.1 Å), and the S^δ^ atom of Met-97 (∼2.6 Å), forming a distorted tetrahedral geometry^34^. Blue-copper proteins owe their name to a strong (∼2.1 eV)^44^ absorption around 600 nm associated with a S_Cys112_, p*π*→Cu ligand to metal charge transfer transition (Supplementary Fig. 14). These particular spectral features are related to the highly covalent nature of the Cu–S bond^45^,^46^, resulting from the strong interaction between the Cu 3d_x_^2^_-y_^2^ acceptor orbital and the 3S p*π* donor orbital^31^,^47^. The high Cu–S stretching frequency of 400 cm^−1^ measured in Resonance Raman experiments supports the strong Cu–S_Cys112_ bond^39^,^48^. S K-edge X-ray absorption spectroscopy results show that ∼38% S character is mixed into the Cu 3d_x_^2^_-y_^2^ orbital in the ground state^39^. Moreover, the highly covalent copper-thiolate bond imparts a narrow four-line hyperfine splitting in the electron paramagnetic resonance spectra^31^. Such strong covalent character notwithstanding, the forces required to break the Cu–S_Cys112_ and the Cu–N_His46_ bonds (Cu–S_Cys89_ and Cu–N_His39_ in plastocyanin) are surprisingly low, of only ∼45 pN (Supplementary Fig. 15). Interestingly, while the forces to unfold plastocyanin are slightly higher (∼75 pN) than those required to unfold azurin (∼55 pN), the mechanical stability of the organocopper bonds in both proteins is ∼45 pN. These values are in sharp contrast with the high mechanical forces required to break homolytic covalent Si–Si bonds (2.1 nN) or heterolytic C–Si (2.0 nN) or Au–S (>2.5 nN) bonds^26^,^27^,^49^. The low mechanical forces required to disrupt the copper-ligand bonds in azurin and plastocyanin, together with the relatively low mechanical stability measured for the Fe–S (∼211 pN; ref. 28) and Zn–S bonds (∼170 pN; ref. 50) in rubredoxin, suggest that, from a mechanical perspective, the labile covalent organometallic bonds are significantly distinct from the mechanically resilient non-metallic covalent bonds such as Si–Si, S–S or Si–C bonds^49^, despite their similar dissociation energies. These experimental findings call for computational efforts aiming at developing novel force fields that couple organometallic bonds with mechanical strain to help elucidation of the quantum-mechanical origin of the force-induced organometallic bond rupture.

The precise novel characterization of the nanomechanical properties of the copper-based organometallic bonds allowed us to uncover the complexity in the azurin and plastocyanin unfolding pathways when placed under mechanical force. In the case of azurin, we attribute the rupture of the unfolding intermediate to the breaking of the Cu–S_Cys112_ and Cu–N_His46_ bonds; however, no obvious intermediate was detected that was compatible with the rupture of the Cu–N_His117_ bond, which has been described using chemical denaturation techniques to be still formed even after azurin unfolding^51^. It is possible that due to the short distance (five residues) within Cys-112 and His-117 within the structure, the length resolution of our instrumentation makes them indistinguishable, being all included in the data defining 6-nm intermediate. However, the observation that for the (Azu_C112A_–I27)_4_ mutant (Fig. 2d) the population of intermediate events at ∼6 nm (or lower) was vanishingly small renders this option unlikely. Hence, it is plausible that on unfolding from the C terminus, the disruption of the β2b–β8 sheets induces a conformational change in the protein loop connecting β7–β8 strands that concomitantly distorts the active site structure thus perturbing the Cu–N_His117_ bond.

**Figure 5:**
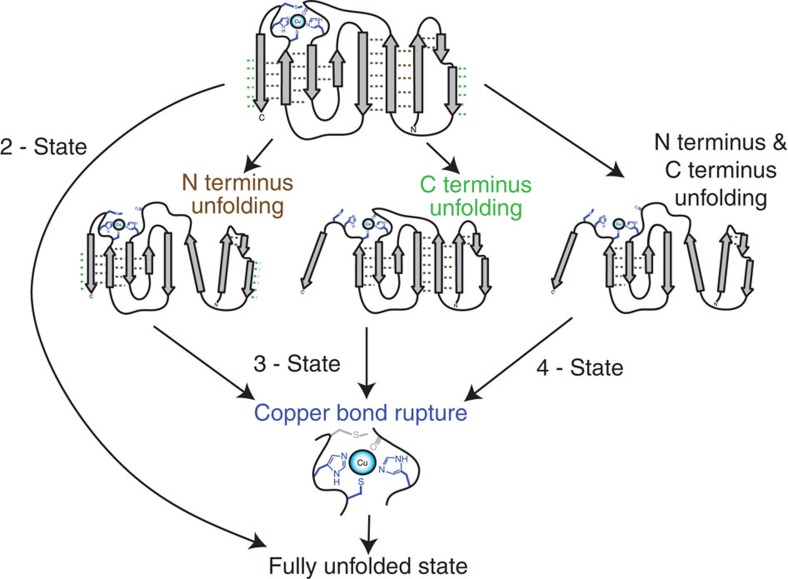
A generic kinetic partition scheme for the complex mechanical unfolding of wt-azurin and wt-plastocyanin. On the application of mechanical force, azurin can unfold following multiple pathways. In 20% of the occurrences (40% in the case of plastocyanin), the protein unfolds according to an all-or-none process. By contrast, in 75% of the cases (50% for plastocyanin), azurin unfolds via one mechanically stable intermediate after unfolding from either the N terminus (brown) or the C terminus (green). In the remainder 5% (10%) of the cases, a four-state unfolding pathway involving the simultaneous unfolding of the N and C termini is observed.

A remarkable finding in our experiments was the stochastic rupture of the main unfolding barrier in both studied blue-copper proteins, azurin and plastocyanin. Due to the particular β-barrel topology of the cupredoxin fold, two independent mechanical transition states, each presumably placed close to both of the termini, govern the mechanical stability of the proteins. Fortuitously, the height of the energy barrier to unfold both termini in each protein was found to be almost equivalent when a pulling velocity of 400 nm s^−1^ was employed, requiring a relatively low force of ∼55 pN to unfold both termini in azurin (Supplementary Fig. 15), and ∼75 pN to unfold both C and N termini in the case of plastocyanin (Fig. 3e). In fact, MD simulations on azurin clearly revealed that for both transition states, the rupture of the hydrogen bonds between the involved β-sheets occurs through an unzipping mechanism because the force is applied parallel to the hydrogen bonds axes (Fig. 4a). The precise dynamics of the unfolding sequence from both protein termini is detailed in the Supplementary Methods. These results put forward a rather unique global mechanical unfolding scenario for wt-azurin and wt-plastocyanin (Fig. 5). In 20% of the cases (40% in the case of plastocyanin), azurin unfolds in a single two-state event. In this case, we cannot pinpoint which mechanical transition state, either the N or C termini, breaks first. It is plausible that in some of these events, the resolution of our instrumentation was not able to detect the fast mechanical intermediates. However, by pulling in force clamp at forces as low as 20 pN (Supplementary Fig. 16), and hence readily expanding the time-span of each trajectory, we could still capture both populations of events; those exhibiting a clear mechanical intermediate and those events where the unfolding was two-state, lacking any measurable intermediate. Indeed, ICP-OES measurements, which precisely measure the copper content in the protein sample, revealed that copper uptake was not total (Supplementary notes). We hence hypothesize that the observed two-state unfolding trajectories correspond to the unfolding of individual apo-azurin (or apoplastocyanin) modules. In 80% of the occurrences, azurin unfolds through a complex pathway involving at least one mechanical intermediate. Of these, in ∼45% of the trajectories mechanical unfolding starts from the C terminus (green pathway), whereas in ∼45% of the cases, unfolding proceeds instead through the N terminus (brown pathway). Finally, in the remainder ∼10% of the occurrences, the protein unfolds first from one of the termini, then from the other. On a few occasions (*n*=16) a unique 15 nm step corresponding to the concomitant unfolding of both termini is observed. Similarly, wt-plastocyanin follows a similar global unfolding scheme, with slightly different percentages corresponding to each unfolding pathway.

The observed similar probability of unfolding occurring from either the C– or the N termini in wt-azurin is severely biased when the native disulfide bond is absent (Fig. 2e). In this case, unfolding mostly occurs (∼80%) from the N terminus. MD simulations further confirm the experimental results by showing that, in 80% of the cases, the reduced wt-Azu lacking its disulfide bond (equivalent to the C26A mutant) unfolds indeed by breaking the N terminus β-sheets. Crucially, in our experiments we observed that wt-azurin can unfold from the N or C termini with almost equal probability. By contrast, our MD simulations show a drastic change in the pathway preference since 90% of the trajectories unfold from the C terminus. It is likely that such discrepancy can be explained by the slightly different distance to the transition state, Δ*x*, associated with the unfolding from both C and N termini, giving rise to distinct force dependencies of unfolding. Since the force range probed in the *in-vitro* and *in-silico* experiments is significantly different, it is plausible that both approaches capture different relative unfolding probabilities at the distinct sampled pulling forces.

Altogether, the whole unfolding cascade is reminiscent of a complex kinetic partition scheme. In the recently reported examples for T4 Lysozyme^18^, Maltose Binding protein^21^ and Leucine Binding Protein^23^ the main unfolding event was unique and always present, while mechanical intermediates further along the unfolding pathway were found to be stochastic and occurring in different positions. In the case of GFP, a bifurcation in the unfolding pathway was observed after the detachment of an N-terminal α-helix, fingerprinted by internal engineered disulfide bonds^24^ or circular permutations^52^. By contrast, azurin and plastocyanin exhibit two distinct mechanical clamps placed very close to each terminus that exhibit a similar stability. Hence, on application of force, the protein stochastically crosses one of the two main barriers to unfolding. The directionality of the entire unfolding pathway is therefore determined by this early random event. Once the barrier is crossed, the protein unravels and gets held ‘on pathway’ at well-defined positions around the copper site. Taken together, the new unfolding scheme depicted for both blue-copper proteins follows a complex kinetic partition scheme in which the stochastic nature of the process is highlighted at the very beginning of the unfolding process with the rupture of the hydrogen bonds determining the main barrier for unfolding.

Our experiments have allowed us to pinpoint the unfolding directionality in the studied blue-copper proteins, occurring either from the C or N termini independently. The choice in the terminus that initiates unfolding might be of biological relevance, as demonstrated by recent nanopore-based experiments, which have successfully recorded the distinct co-translocational unfolding dynamics depending on the terminus that is first presented to the nanopore mouth^53^. Similarly, during degradation in the proteasome, proteins are mechanically unfolded before entering the narrow proteolytic chamber^54^,^55^. The direction of entry in both processes depends on the force needed to unfold its two termini^56^. When the N and C termini are mechanically non-equivalent, the protein will presumably tend to search for the weak link to initiate unfolding at the pore mouth^57^. In this regard, proteins with dual mechanical transition states might provide an interesting case study for these applications, especially if one of the N or C termini can be tuned at will to act as the protein’s Achilles’ heel^58^.

Altogether, our experiments demonstrate the power of single molecule techniques at revealing complex unfolding pathways in topologically rich proteins such as the case of metalloproteins, of high relevance in nature.

## Methods

### Protein engineering

The (Azu–I27)_4_, (Azu_C112A_–I27)_4_, (Azu_C26A_–I27)_4_ and (Plasto-I27)_4_ polyproteins were subcloned using the BamHI, BglII and KpnI restriction sites^59^. Synthetic gene for plastocyanin was produced by Life Technologies (Paisley, UK). The multidomain proteins were cloned into the pQE80L (Qiagen) expression vector, and transformed into the BLR(DE3) *Escherichia coli* expression strain. All mutations were introduced by using the QuikChange Site-Directed Mutagenesis kit (Agilent). The cells were grown in LB broth supplemented with 100 μg ml^−1^ ampicillin at 37 °C. After reaching an OD_600_ of ∼0.6 the cultures were induced with Isopropyl β-D-1-thiogalactopyranoside (1 mM) and incubated overnight at 20 °C (constructs containing Azurin) or 16 °C (Plastocyanin construct). For some constructs, CuSO_4_ (0.5 mM) was supplemented to the cultures right before induction to increase copper intake. After harvesting the cells, disruption using a French Press was performed. The polyproteins from the lysate were purified by metal affinity chromatography on Talon resin (Clontech) followed by gel-filtration using a Superdex 200 10/300 GL column (GE Biosciences). Protein concentration in the samples was estimated using the Bradford protein assay.

Details on the biochemistry characterization assays regarding circular dichroism, Ellman’s assays and ICP-OES measurements are detailed in the Supplementary Methods Section.

### Force spectroscopy

Constant velocity AFM experiments were conducted at room temperature using both a home-made set-up^60^ and a commercial Luigs and Neumann force spectrometer^61^. In all cases, the sample was prepared by depositing 1–10 μl of protein in PBS solution (at a concentration of 1–10 mg ml^−1^) on a freshly evaporated gold coverslide. Each cantilever (Si_3_N_4_ Bruker MLCT-AUHW) was individually calibrated using the equipartition theorem, giving rise to a typical spring constant of ∼12–35 pN nm^−1^. Single proteins were picked up from the surface and pulled at a constant velocity of 400 nm s^−1^. Experiments were carried out in a sodium phosphate buffer solution, specifically, 50 mM sodium phosphate (Na_2_HPO_4_ and NaH_2_PO_4_), 150 mM NaCl, pH=7.2.

### Data analysis

All data were recorded and analysed using the custom software written in Igor Pro 6.0 (Wavemetrics). For all polyproteins, only recordings showing the signature of at least two events corresponding to the unfolding of the I27 fingerprints were analysed. Statistical tests were performed by applying Hartigan’s diptest statistic for unimodality/multimodality^62^,^63^ and provided a test with simulation based *P* values based on a package compiled in R. Information about the data supporting this research and conditions of access can be found by emailing research.data@kcl.ac.uk.

### MD simulations

Constant-force steered MD were performed on azurin starting from the PDB structure 4AZU solvated in a water box large enough in the direction of pulling to accommodate the partially unfolded protein (6 × 6 × 18 or 6 × 6 × 21 nm). The NAMD 2.10 software was used to run the simulations on pure CPU or CPU/GPU nodes. Atomic parameters correspond to those in the CHARMM36 force-field^64^, while atomic charges for copper II and atoms of the five ligands were taken from quantum calculations^65^. We also constrained the organometallic bond distances as described elsewhere^65^. Starting from a previously equilibrated protein whose CN axis was aligned along the longer edge of the box (direction of pulling), the C_α_ atom of the first residue was fixed while a constant force (250, 310 or 350 pN) was applied to the C_α_ atom of the last residue. In half of the trajectories, C_α_ from residue no. 1 was fixed and C_α_ from residue no. 128 was pulled; for the other half, C_α_ of residue no. 128 was fixed and C_α_ from residue no. 1 was pulled in the opposite direction. Twenty-one such trajectories, lasting between 6 and 20 ns (until full possible extension was reached), were generated for the protein containing the disulfide bond (10 or 11 in each direction) in a ∼18-nm long box; nine trajectories total were generated in the absence of disulfide bond, in a slightly larger box (∼21 nm). A very similar methodology was employed to simulate plastocyanin (3BQV, *n*=10 trajectories—5 in each direction) and apoplastocyanin (2PCY, *n*=10 trajectories—5 in each direction). As the active site of plastocyanin is very similar to that of azurin, we used the same set of atomic charges and bond potentials, albeit at slightly different equilibrium distances corresponding to that measured in the crystal structure.

## Additional information

**How to cite this article:** Beedle, A. E. M. *et al*. The mechanochemistry of copper reports on the directionality of unfolding in model cupredoxin proteins. *Nat. Commun*. 6:7894 doi: 10.1038/ncomms8894 (2015).

## Supplementary Material

Supplementary Information Supplementary Figures 1-16, Supplementary Tables 1-7, Supplementary Note 1, Supplementary Methods and Supplementary References
