# Supplementary material for: The mechanochemistry of copper reports on the directionality of unfolding in model cupredoxin proteins
Source: Nat Commun. 2015 Aug 3;6:7894. doi: 10.1038/ncomms8894 (PMC4532836; doi:10.1038/ncomms8894)
Supplement: Supplementary Information — Supplementary Figures 1-16, Supplementary Tables 1-7, Supplementary Note 1, Supplementary Methods and Supplementary References [file ncomms8894-s1.pdf]

Supplementary Information

The mechanochemistry of copper reports on the directionality of unfolding in model cupredoxin proteins

Amy E.M. Beedle[1], Ainhoa Lezamiz[2], Guillaume Stirnemann[3] and Sergi Garcia-Manyes[1,2*]

[1]Department of Physics and [2]Randall Division of Cell and Molecular Biophysics, King's College London, WC2R 2LS, London, UK.
[3] CNRS Laboratoire de Biochimie Théorique, Institut de Biologie Physico-Chimie, Paris, 75005, France

Correspondence: S.G.-M. (sergi.garcia-manyes@kcl.ac.uk)

Supplementary

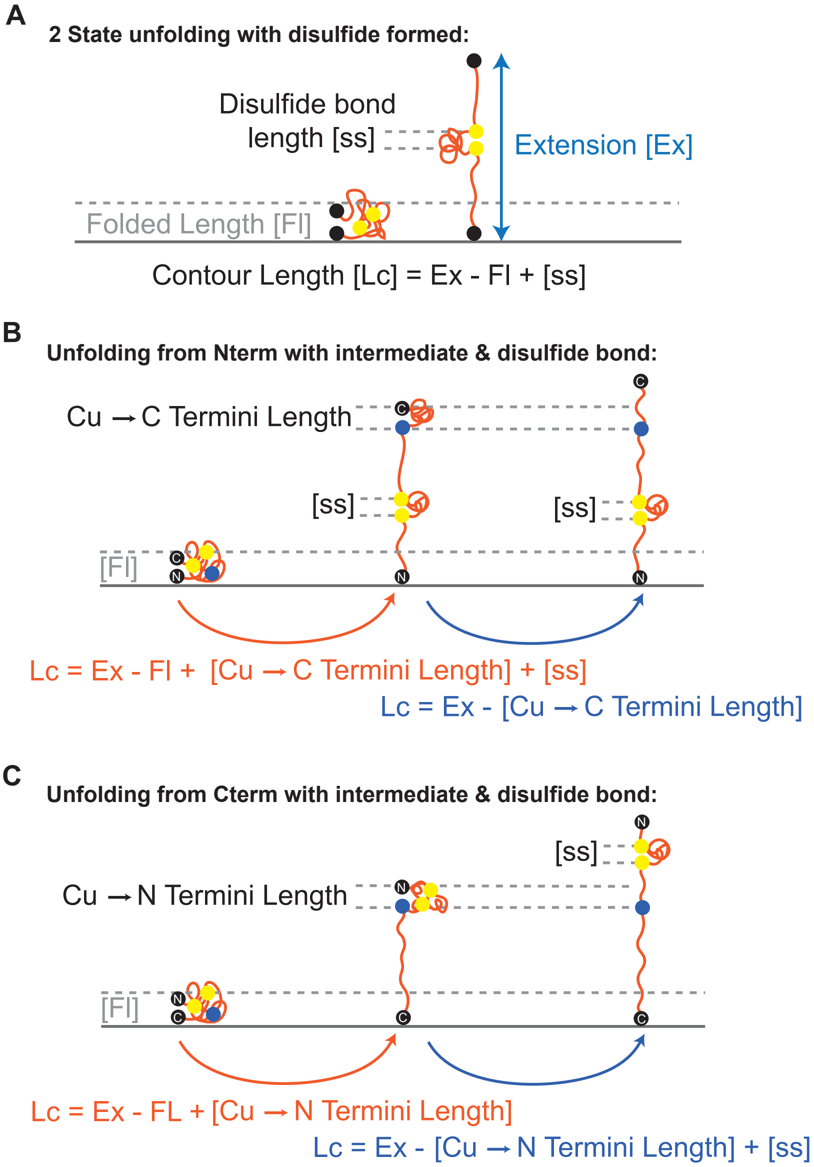

Figures

Supplementary Figure 1. Schematic representation of the two- and three-state (via N- or C- terminus) unfolding of a single azurin molecule containing a disulfide bond. The corresponding equations to calculate the increment in contour length for each event are provided. (A) When no intermediate is observed, the expected increment in contour length (L_C) is calculated by subtracting the initial folded length (Fl = 1.12 nm, which is the distance between the N- and C- termini obtained from the crystal structure) to the protein extension, (Ex = # of amino acids * 0.38 nm/aa – Fl] and adding the length of the formed disulfide bond (ss). (B) When the unfolding process occurs via an intermediate, the contribution of the disulfide bond length is dictated by which termini unfolds. In addition, the remaining folded portion of the protein must be accounted for. As the disulfide bond is located between the N-terminus and His-46, when the N-terminus unfolds the bond length is accounted for in the initial unfolding event (orange equation). (C) Conversely, when the C-terminus

unfolds first, the disulfide bond remains in the folded region of the protein, and is only extended once the metallo-bond has been ruptured (blue equation).

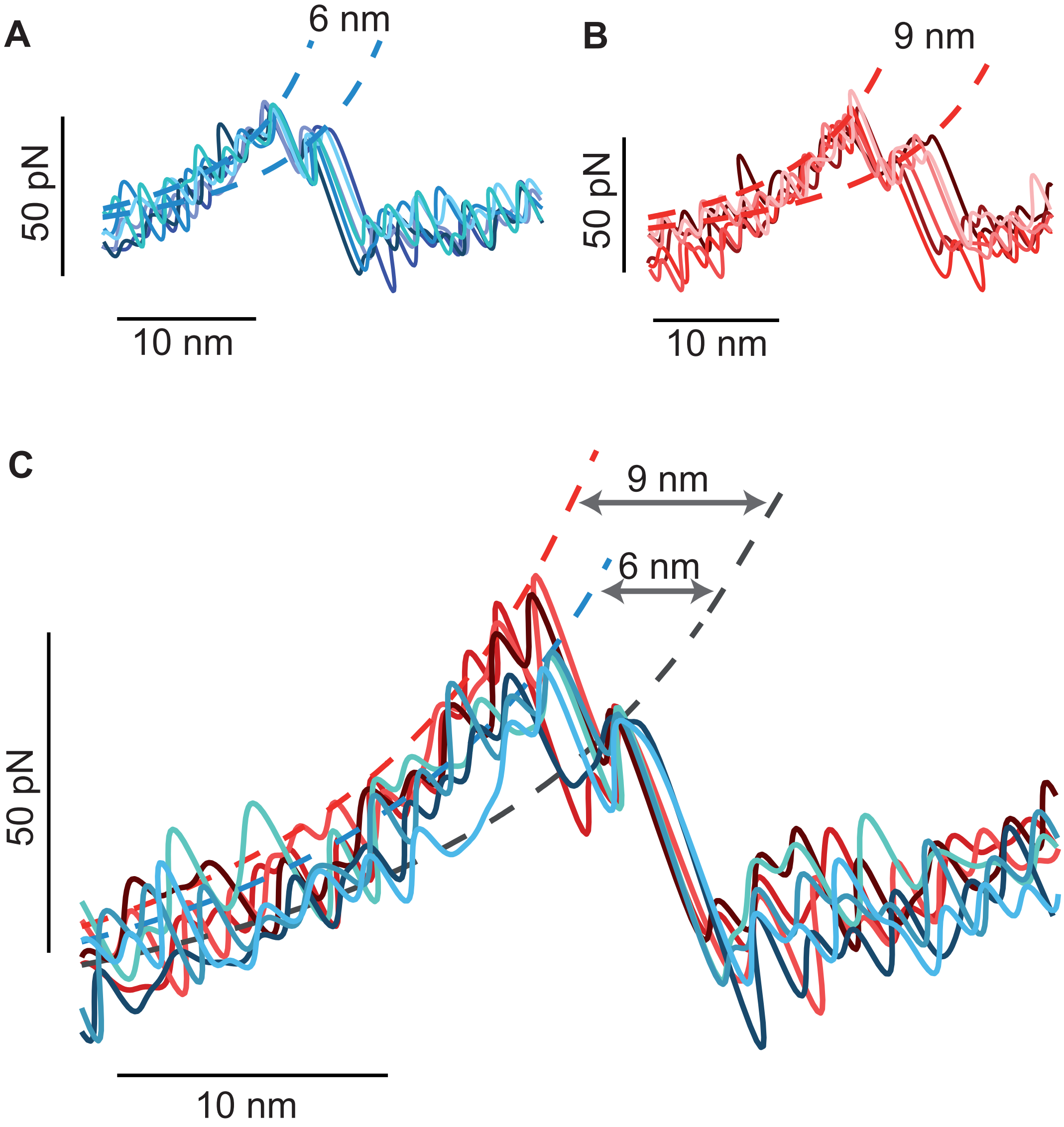

Supplementary Figure 2. Comparison of multiple azurin unfolding events containing a single intermediate (A) Multiple trajectories displaying a ~6nm (dashed blue WLC fit) corresponding to the C-terminus unfolding up to the C112-Cu bond (*n*=6). (B) Overlay of *n*=6 events where azurin unfolding occurs from the N-terminus, extending the protein by ~9nm up to the Cu-N_{His46} bond (dashed red WLC fit, *n*=6). (C) Direct comparison of the increment in contour length associated to the unfolding from either the N-terminus (red), or the C-terminus (blue). In the representation, each trajectory is aligned at the point of metallobond rupture.

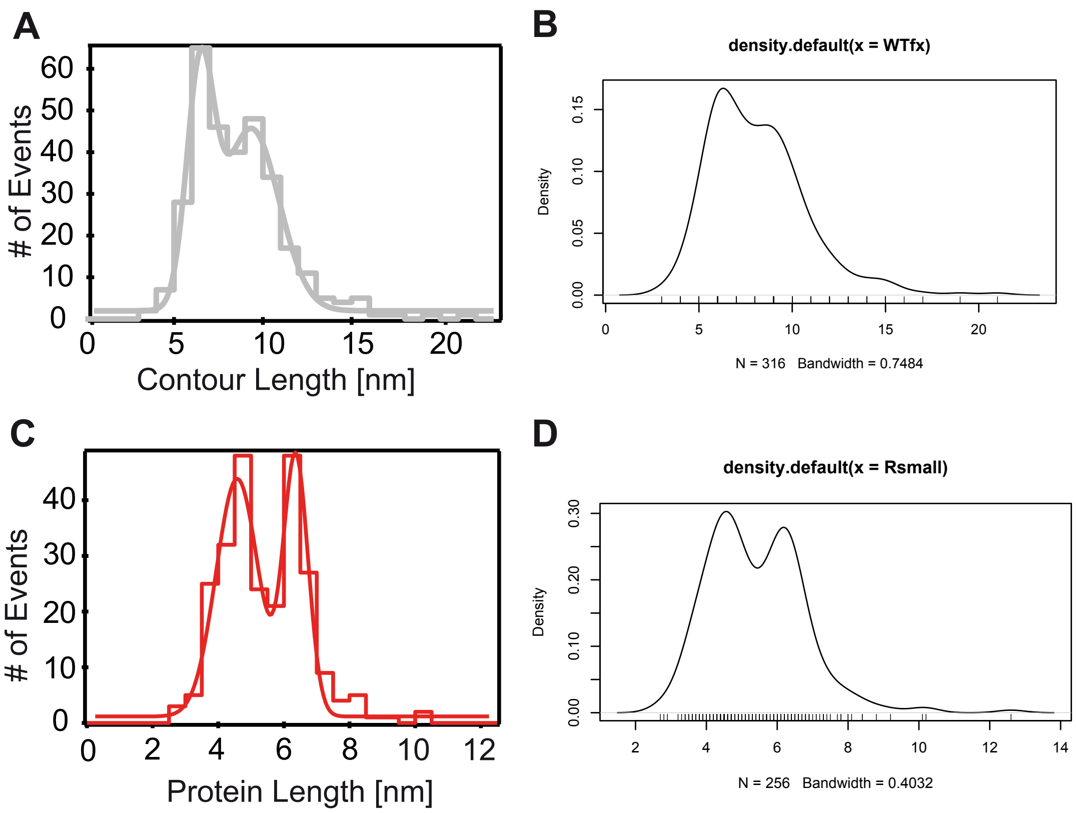

Supplementary Figure 3. The distribution of the increment in contour length for the different intermediate states measured in both force-extension and force-clamp experiments is multimodal, as revealed by the Hartigans' diptest. (A) Histogram corresponding to the increment in contour length for the different intermediate states measured under constant velocity conditions (Fig. 1F and 2D). (B) Applying Hartigans' diptest to force extension data results in *D*=0.0759, *p* <2.2 e^{-16}, thus statistically proving the alternative hypothesis (i.e. non-unimodal, at least bimodal) distribution of data. (C) Multi(bi)modality in the histogram of step sizes that correspond to the unfolding of the protein up to mechanical intermediate measured under force clamp conditions (Supplementary Figure 4C) is demonstrated (D) by the results from the Hartigans' diptest, *D* =0.0511, *p* = 4.62 e^{-5}. Hartigan's diptest statistic for unimodality/multimodality provided with a test with simulation-based *p*-values was applied thanks to a package compiled in R (http://cran.rproject.org/web/packages/diptest/diptest.pdf).

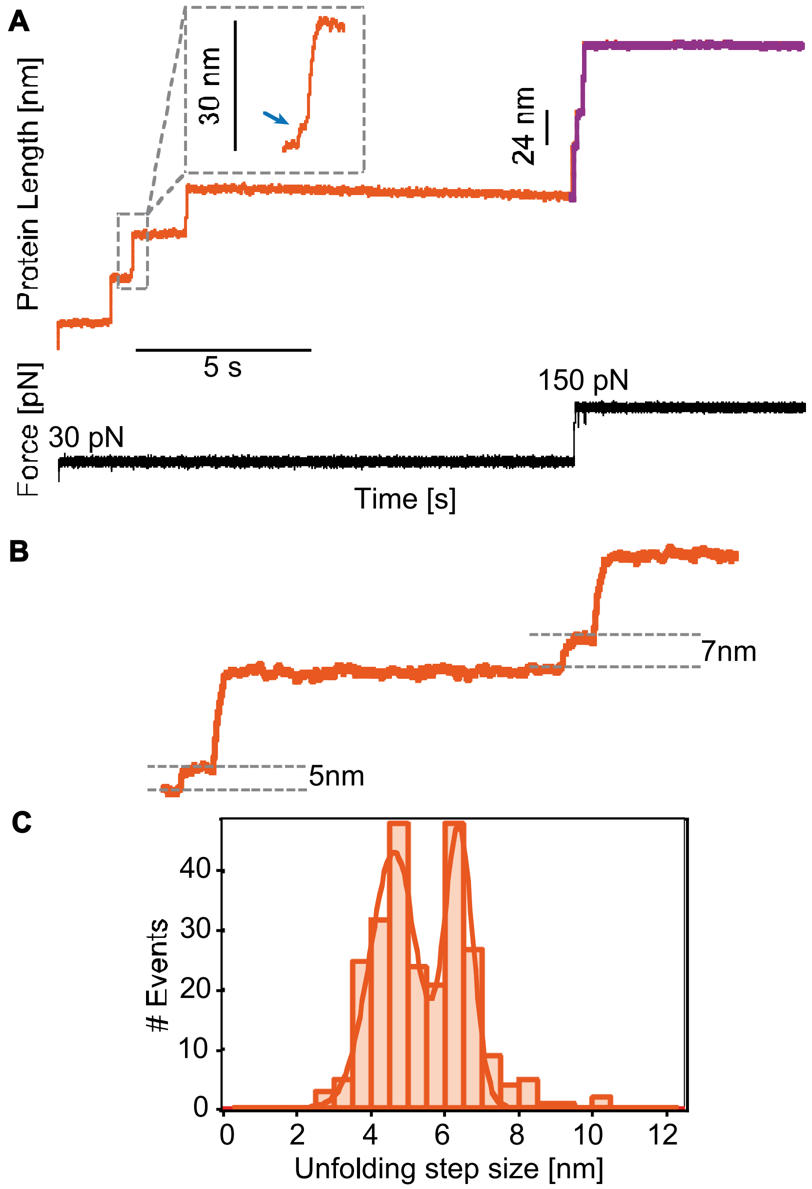

Supplementary Figure 4. Force-clamp experiments further confirm the presence of two distinct mechanical intermediates. (A) Typical unfolding trajectory of the (Azu-I27)$_4$ polyprotein. Using a two force-protocol, a constant force of 30 pN was first applied for 15 seconds, which triggered the unfolding of the individual azurin modules (~29 nm steps), often occurring via a fast, mechanical intermediate (blue arrow, *inset*). A second pulse at 150 pN promotes the unfolding of the mechanically resistant I27 fingerprints (~24.5 nm steps), ensuring the single molecule nature of the experiment. (B) Unfolding trajectory of two consecutive azurin monomers occurring via a ~4nm and a ~6 nm step mechanical intermediate, respectively. (C) Measurement of the distribution of step lengths corresponding to the unfolding of n = 256 individual azurin modules up to the mechanical intermediate. The resulting histogram is bimodal with a 99.99% certainty (Hartigan's statistical diptest), thus confirming the presence of two independent and distinct intermediates centred at ~4.3 ± 0.6 nm and ~ 6.1 ± 0.4 nm in the mechanical unfolding of wt-azurin.

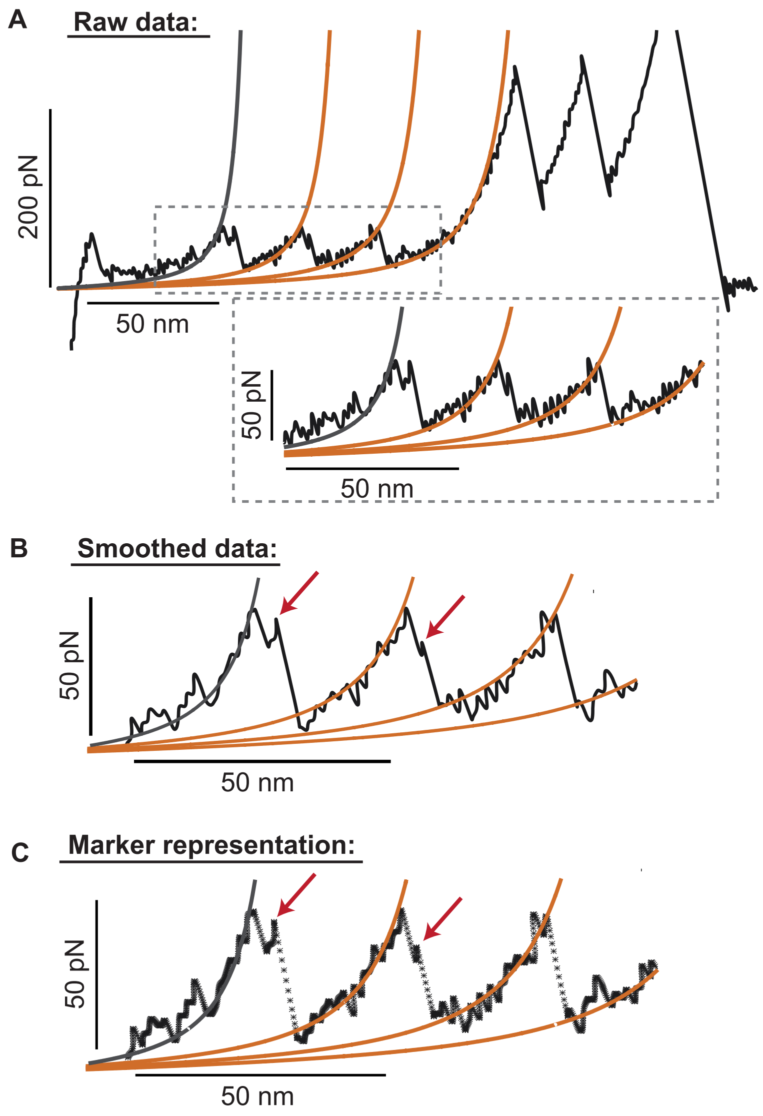

Supplementary Figure 5. Detection method for intermediate events in the unfolding trajectory of a single molecule in force extension mode. (A) Raw data trajectory of three azurin unfolding events (Orange WLC) followed by the unfolding of two I27 marker proteins. Trajectories are typically acquired using a high Nyquist fraction (~0.5) and a high number of points per trace (~5,000-9,000). (B) Reducing the Nyquist fraction down to (0.1) and applying a low pass filter, which removes small fluctuations to reveal the unaltered form of the data, provides further evidence of the presence of a clear mechanical intermediate (red arrow) that occurs after the main protein unfolding event. (C) Representing the trajectory as markers instead of a continuous line, where each marker corresponds to an acquired data point, reveals a high density of data points defining the intermediate. The localisation of data points indicates that the cantilever gets temporarily 'held' at a mechanically resilient event, signature of barrier crossing. In the case of azurin, such mechanical resilience originates from one of the metallobonds coordinating the copper ion.

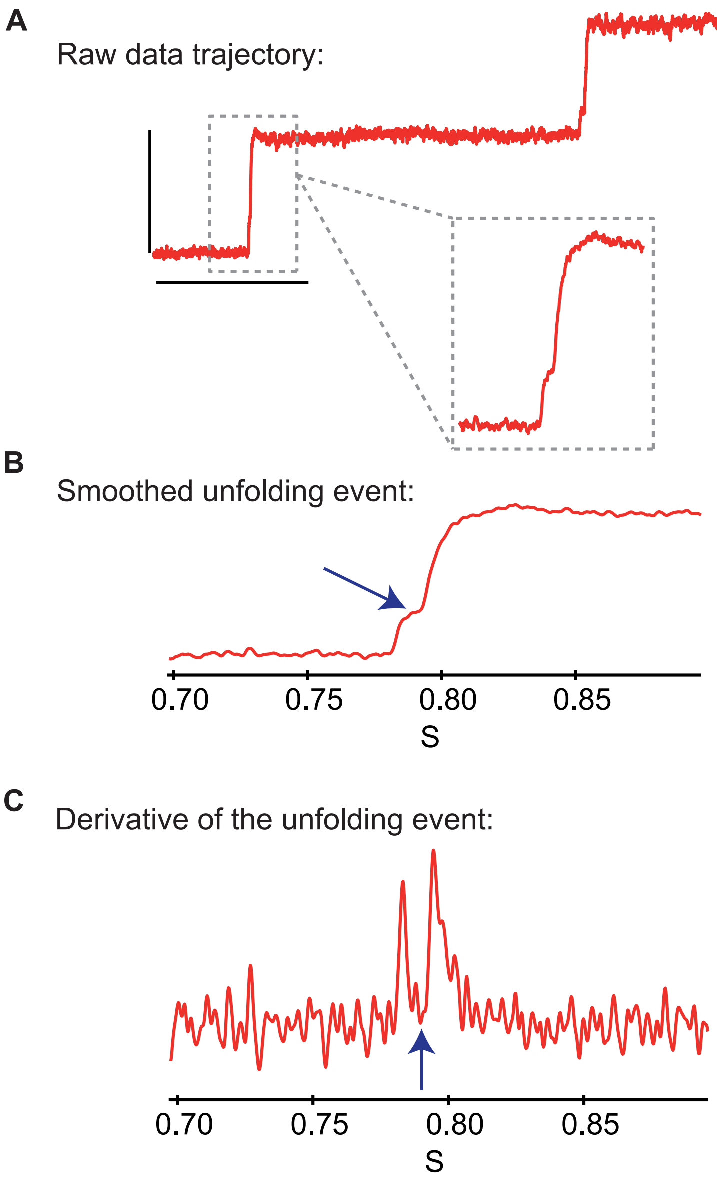

Supplementary Figure 6. Detection method for the unfolding intermediates observed in force clamp spectroscopy. (A) Raw single molecule force clamp trajectory displaying the unfolding of two consecutive wt-azurin monomers at a constant force of 30pN. Within the main unfolding step, an intermediate corresponding to the rupture of the metallobond can also be observed. In some cases the intermediate occurs quickly (~6 ms, dashed inset). (B) In order to unambiguously determine the step height in these instances, we firstly smooth the trajectory by applying a 20-point box filter, which plots the average point of the neighbouring 20 data points. (C) We then took the derivative of the filtered length trace to highlight the point of inflection, which provides precise location of the intermediate event.

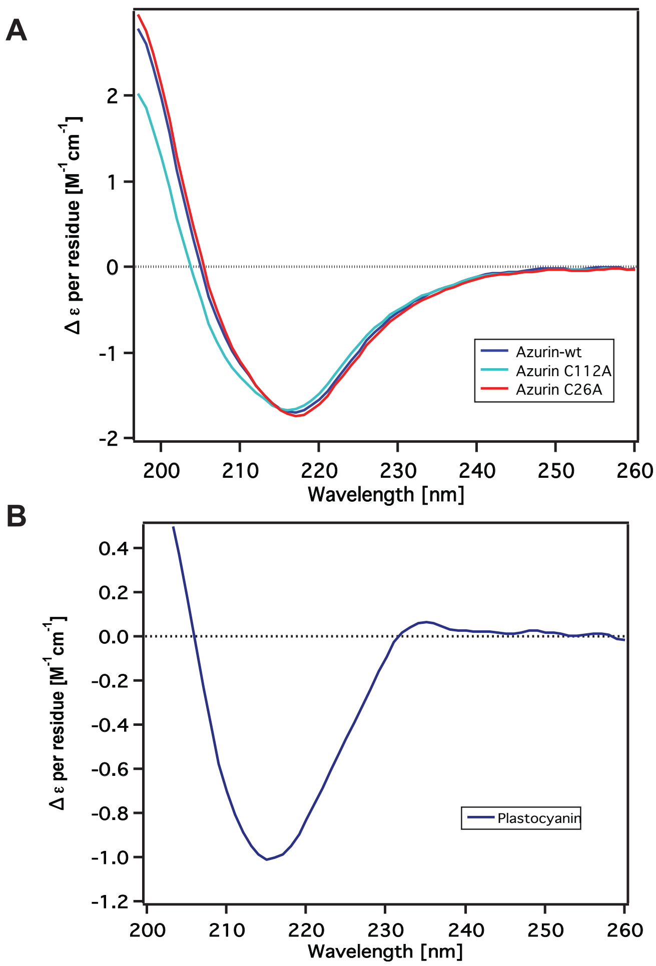

Supplementary Figure 7. CD spectra of the different polyproteins reveal that in all cases the protein is correctly folded. (A) CD spectra comparing the folding of the three $(Azu\text{-}I27)_4$, $(Azu_{C112A}\text{-}I27)_4$, $(Azu_{C26A}\text{-}I27)_4$ polyproteins used in our single molecule experiments. Crucially, the spectrum of the Azu_{C112A} mutant displays a similar behaviour as the Azu_{wt} and Azu_{C26A} forms, suggesting that the protein is correctly folded. (B) Similarly, the CD spectrum of the $(Plasto\text{-}I27)_4$ polyprotein ensures the correct folding of the wt-plastocyanin protein.

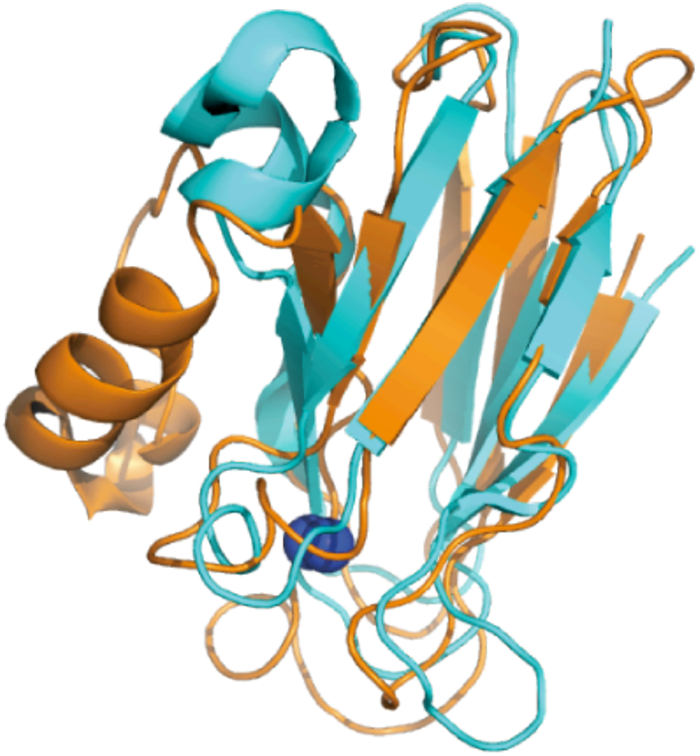

Supplementary Figure 8. Structural similarity between plastocyanin and azurin. Alignment of the azurin (PDB:1AZU, orange) and plastocyanin (PDB:3BQV, cyan) structures highlights the similar topology of the two proteins, only differing in the length of the alpha helix (encompassing 23 amino acids) that is present in azurin but absent in plastocyanin.

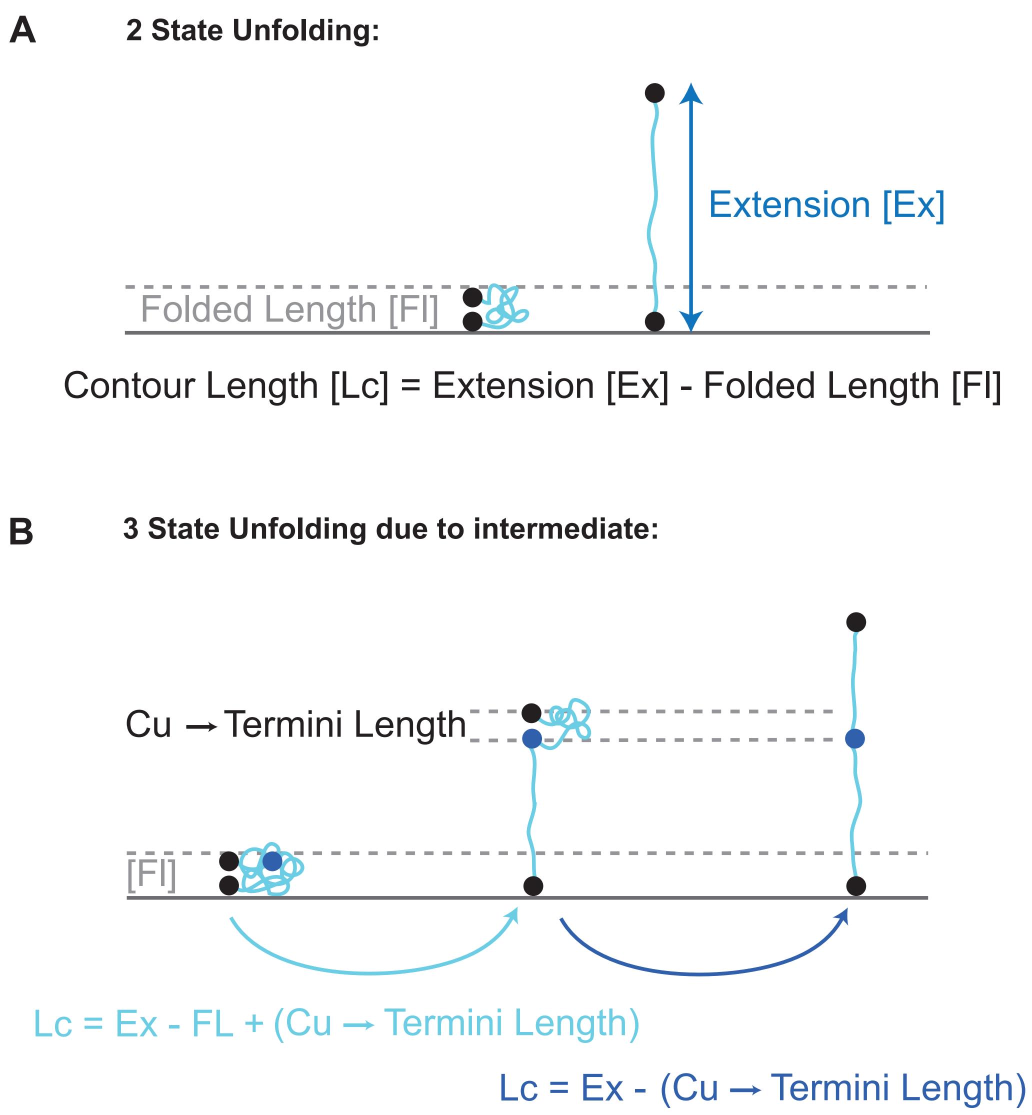

Supplementary Figure 9. Schematic representation of the calculated increment in contour lengths for the unfolding of a single plastocyanin molecule. (A) When unfolding occurs in a two-state process, the calculated increment in contour length simply corresponds to the protein extension [Ex, given by number of amino acids * 0.38nm/aa - the folded length (Fl) of the protein]. The folded length (Fl = 1.53 nm) is the distance between the N- and C-termini obtained from the crystal structure. (B) When an intermediate is observed, the equation shown in (A) must still hold true between the first and final event, however the folded length between the copper and the remaining folded protein must be accounted for. The light blue equation gives the increment in contour length for termini unfolding up to the copper, and the

equation in dark blue gives the remaining length once the copper-bond has ruptured.

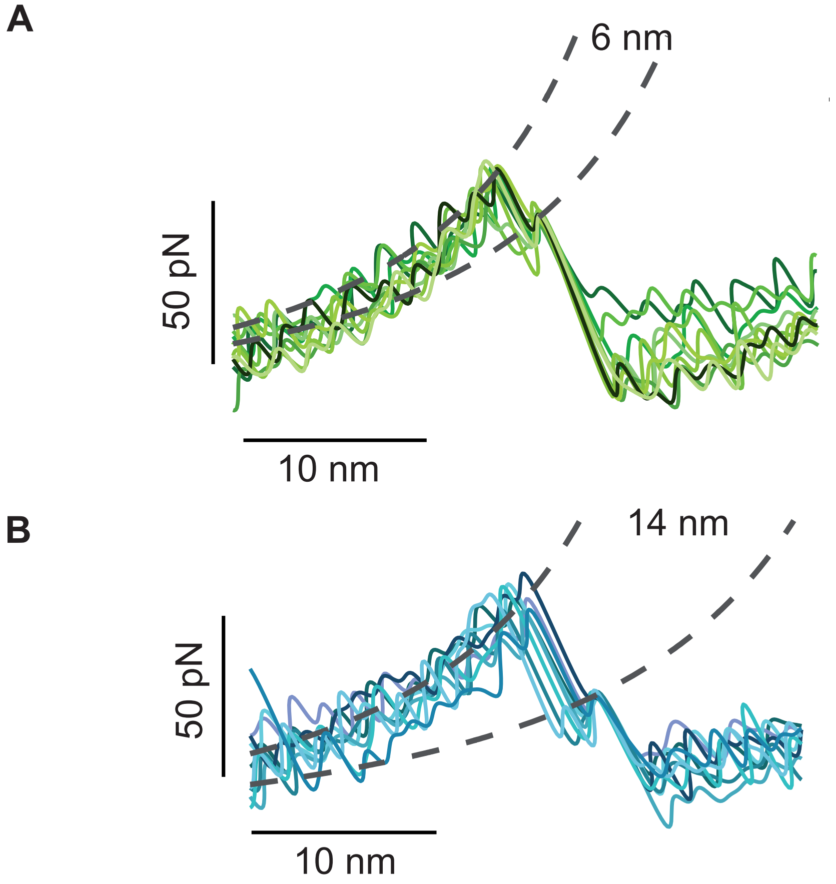

Supplemenary Figure 10. Plastocyanin displays two readily distinct mechanical intermediates. (A) The overlay of *n*=10 plastocyanin unfolding events containing a clear mechanical intermediate of ~6nm, corresponding to the unfolding of the C- terminus up to the Cys-89 or His92 – Cu bond. (B) Similarly, unfolding from the N-terminus up to the mechanically resilient His39 copper bond corresponds to an increment in contour length of ~14nm (WLC fit, grey dashed lines, *n*=9).

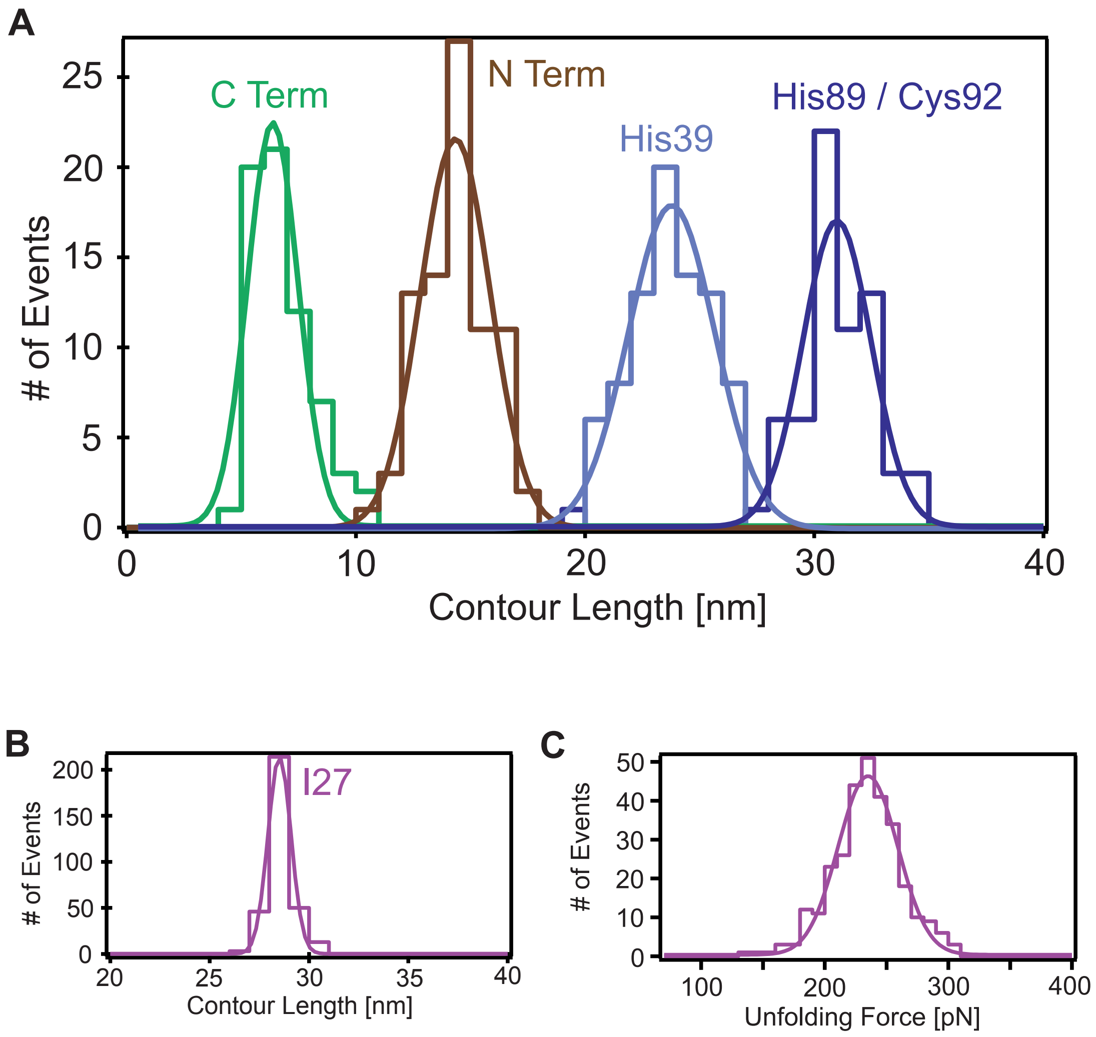

Supplementary Figure 11. Increment in contour length hallmarking the intermediates encountered upon unfolding plastocyanin from the N- and C-termini. (A) Histogram of contour lengths corresponding to the protein unfolding from the N-terminus up to the His39–Cu bond (brown, 13.8 ± 1.4 nm, *n*=82) and to the remaining extension once the bond has ruptured up to the total protein contour length (light blue, 23.3 ± 1.9 nm, *n*=82). Similarly, when the C-terminus unfolds up to either the Cys89 or His92–Cu bond, an increment of 6nm is obtained (green, 5.9 ± 1.1 nm *n*=66). Once this bond has been ruptured, the remaining 30.5 ± 1.5 nm is measured (dark blue, *n*=66). (B) Histogram of increment in contour length for the I27 marker protein (28.0 ± 0.6 nm, *n*=326) and (C) its corresponding unfolding force (230 ± 24 pN , *n*=326).

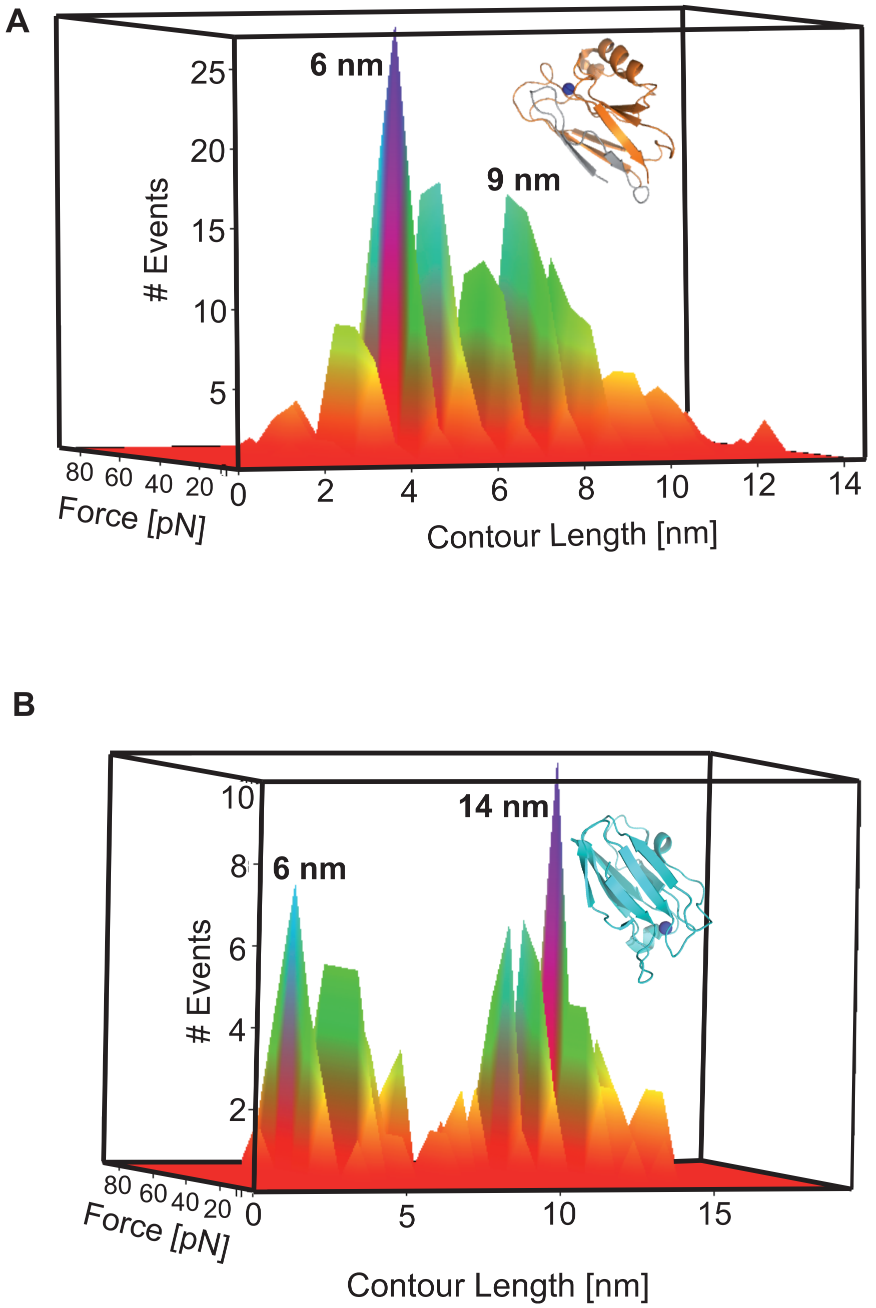

Supplementary Figure 12. 3-dimensional histogram of contour length increment and unfolding force corresponding to mechanical unfolding of wt-azurin and wt-plastocyanin. (A) A 3D histogram for the initial unfolding events of the wt-azurin. Two distinct population of events, occurring at ~6nm and ~9nm, are observed. (B) For plastocyanin, the two populations of events is also observed. Due to the geometry of the copper binding site and the protein topology, the ~6nm intermediate (corresponding to C-terminus unfolding) and the ~14 nm intermediate (corresponding to unfolding from the N-terminus) are in this case further separated in terms of increment in contour length.

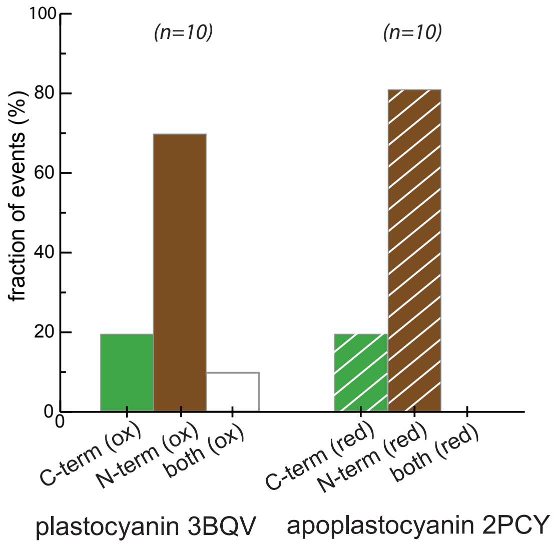

Supplementary Figure 13. Probability of plastocyanin unfolding from the N-term and C-term for both the wild-type and apo-plastocyanin forms in our steered molecular dynamics simulation. The almost identical probability of unfolding brom both termini suggests that the metallic centre apparently has none (or very little) influence on the first steps of mechanical unfolding, involving the rupture of key hydrogen bonds.

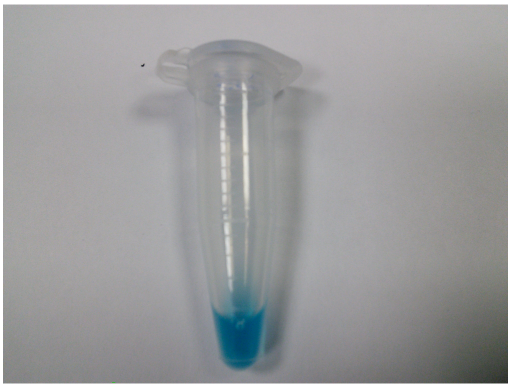

Supplementary Figure 14. Blue copper proteins owe their name to the strong 600 nm absorption band associated to the S_{Cys}, pπ → Cu charge transfer transition. After talon resin purification, the wt-azurin polyprotein used in our experiments exhibits an intense blue colour.

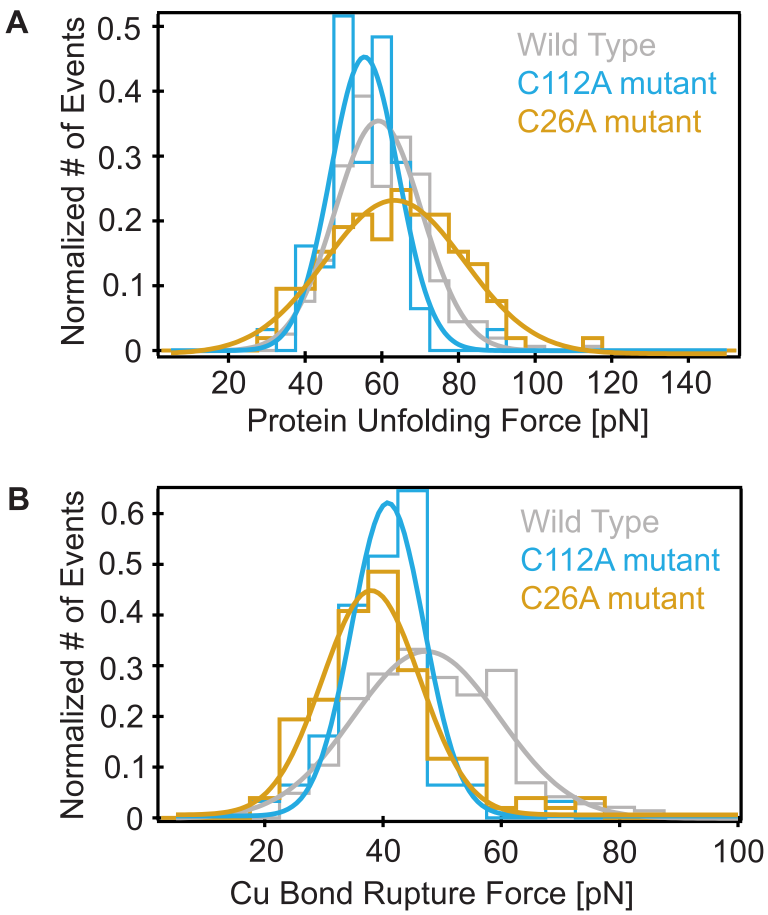

Supplementary Figure 15. The unfolding forces are identical for the three (Azu-I27)$_4$, (Azu$_{C112A}$-I27)$_4$ and (Azu$_{C26A}$-I27)$_4$ polyproteins. Similarly, the measured forces required to disrupt the intermediate conformations upon breaking individual Cu-S or Cu-N bonds are almost identical for all three constructs. (A) Gaussian fit to the distribution of the forces required to unfold the wt-Azu protein (grey) yields a value of 54.1 ± 11 pN, *n* = 320. The values obtained for the unfolding of the Azu$_{C112A}$ (blue) and Azu$_{C26A}$ (yellow) polyproteins exhibit similar unfolding forces of 50.5 ± 8.8 pN, *n* = 62 and 58.3 ± 18 pN, *n* = 105, respectively. (B) Similarly, the force required to disrupt the mechanical intermediates is also almost identical in all cases (wt-Azu protein, grey, 42.3 ± 12 pN, *n* = 289; Azu$_{C112A}$, blue, 35.9 ± 6 pN, *n* = 62; Azu$_{C26A}$, yellow, 35.5 ± 8 pN, *n* = 105). Surprisingly, the rupture forces are almost indistinguishable in the case of the rupture of the Cu-S or Cu-N bond.

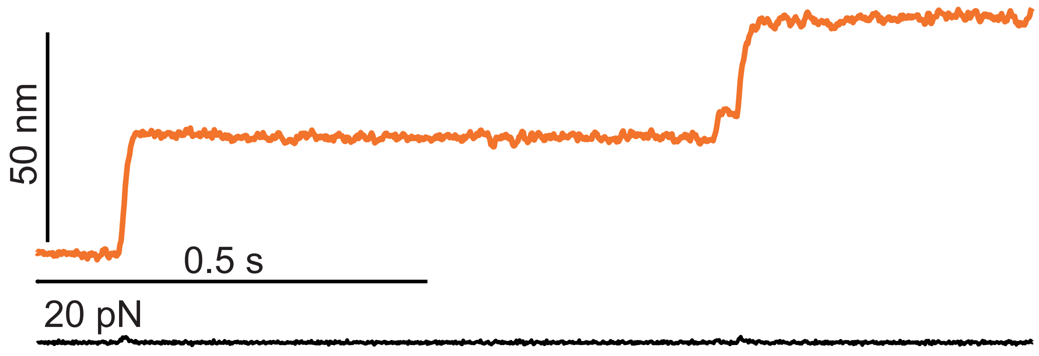

Supplementary Figure 16. Force clamp experiments further demonstrate a complex unfolding pathway in the mechanical unfolding of azurin; while some events occur in a simple two-state scenario, other events exhibit well-defined mechanical intermediate conformations. Pulling an individual (Azu_{C112A}-I27)$_4$ polyprotein at forces as low as 20 pN slows down the unfolding rate and enables the time-separation of the unfolding events. While the first unfolding event occurs in an all-or-none process, the second event exhibits a well-defined intermediate at ~7 nm, corresponding to the ~9 nm event observed under constant-velocity conditions.

Supplementary Tables

Supplementary Table 1

Azurin (PDB:1AZU) – Formed disulfide bond

Total number of amino acids : 105 (= 128 – 23aa trapped behind bond)
Folded length : 1.12nm
N-terminus → Cu length : 2.30 nm
C-terminus → Cu length : 2.76 nm
Disulfide bond length : 0.67 nm

**Whole Protein (2 state unfolding)**		
Expected Length	Measured Length	Measured Force
39.45 nm<br>(105*0.38) – 1.12 + 0.67	38.6 ± 1.0 nm<br>(*n* = 94)	52.7 ± 12 pN
**N Terminus unfolding → His46**		
11.1 nm<br>(23*0.38) - 1.12 + 0.67 + 2.76		
**His46 → Fully unfolded**		
28.4nm<br>(82*0.38) – 2.76		
**C Terminus unfolding → Cys112**		
7.3 nm<br>(16*0.38) - 1.12 + 2.30		
**Cys112 → Fully unfolded**		
32.2 nm<br>(89*0.38) – 2.30 + 0.67		

Azurin – Without disulfide bond

**Whole protein (2 state unfolding)**		
Expected Length	Measured Length	Measured Force
47.5 nm<br>(128*0.38) – 1.12	47.6 ± 1.2 nm<br>(*n* = 77)	56.8 ± 15 pN
**N Terminus → His46**		
19.1 nm<br>(46*0.38) – 1.12 + 2.76		
**His46 → Fully unfolded**		
28.4 nm<br>(82*0.38) – 2.76		
**C Terminus → Cys112**		
7.3 nm<br>(16*0.38) – 1.12 + 2.30		
**Cys112 → Fully unfolded**		
40.3 nm<br>(112*0.38) – 2.30		

Supplementary Table 1. Calculated increment in contour lengths for the unfolding of a single azurin molecule for all encountered unfolding scenarios (both with and without a formed disulfide bond), based on the schematics shown in Supplementary Figure 1.

Supplementary Table 2

Plastocyanin (PDB:3BQV)

Total number of amino acids : 105
Folded length : 1.53nm
N-terminus → Cu length : 2.43 nm
C-terminus → Cu length : 2.94 nm

**Whole Protein (2 state unfolding)**		
Expected length	Measured Length	Measured Force
38.4 nm<br>(105*0.38) – 1.53	36.8 ± 1.1 nm<br>(*n* = 135)	75.8 ± 12 pN
**N Terminus unfolding → His39**		
16.23 nm<br>(39*0.38) - 1.53 + 2.94	13.8 ± 1.5 nm<br>(*n* = 82)	75.3 ± 12 pN
**His39 → Fully unfolded**		
22.1nm<br>(66*0.38) – 2.94	23.3 ± 1.9 nm<br>(*n* = 82)	46.0 ± 11 pN
**C Terminus unfolding → Cys89**		
7.0 nm<br>(16*0.38) - 1.53 + 2.43	5.9 ± 1.1 nm<br>(*n* = 66)	65.9 ± 13 pN
**Cys89 → Fully unfolded**		
31.4 nm<br>(89*0.38) – 2.43	30.5 ± 1.5 nm<br>(*n* = 66)	51.8 ± 13 pN

Supplementary Table 2. Calculated increment in contour lengths for the unfolding of a single plastocyanin molecule in all measured unfolding scenarios, based on the schematics shown in Supplementary Figure 9.

Supplementary Table 3

Simulation ID	Force (pN)	Pulling direction	Unfolding time (ns)	Which side?
1	250	C to N	10	both
2	250	C to N	6	C term
3	350	C to N	4	N term
4	350	C to N	2	C term
5	350	C to N	2	C term
6	250	C to N	2.5	C term
7	250	C to N	2.5	C term
8	250	C to N	2	C term
9	250	C to N	2.5	C term
10	250	C to N	3.5	C term
11	250	C to N	3	C term
12	250	N to C	8	C term
13	310	N to C	2	C term
14	250	N to C	7	C term
15	350	N to C	1.5	C term
16	250	N to C	2	C term
17	250	N to C	2	C term
18	350	N to C	1	C term
19	350	N to C	1	C term
20	310	N to C	5	C term
21	310	N to C	5	C term

Supplementary Table 3. Description of each individual MD simulation run corresponding to wt-Azu unfolding.

Supplementary Table 4

Simulation ID	Force (pN)	Pulling direction	Unfolding time (ns)	Which side?
1	250	N to C	10	N term
2	350	N to C	1	N term
3	350	N to C	4	N term
4	310	N to C	6	both
5	310	N to C	6	both
6	310	C to N	2	N term
7	310	C to N	2	N term
8	350	C to N	1	N term
9	250	C to N	7	N term

Supplementary Table 4. Description of each individual MD simulation run corresponding to the unfolding of wt-Azu lacking its disulfide bond.

Supplementary Table 5

Simulation ID	Force (pN)	Pulling direction	Which side?
1	250	N to C	both
2	310	N to C	C term
3	310	N to C	N term
4	310	N to C	C term
5	350	N to C	N term
6	250	C to N	N term
7	310	C to N	N term
8	310	C to N	N term
9	350	C to N	N term
10	350	C to N	N term

Supplementary Table 5. Description of each individual MD simulation run corresponding to wt-plastocyanin unfolding.

Supplementary Table 6

Simulation ID	Force (pN)	Pulling direction	Which side?
1	250	N to C	N term
2	250	N to C	N term
3	250	N to C	C term
4	350	N to C	N term
5	350	N to C	C term
6	250	C to N	N term
7	250	C to N	N term
8	250	C to N	N term
9	350	C to N	N term
10	350	C to N	N term

Supplementary Table 6. Description of each individual MD simulation run corresponding to wt-apo-plastocyanin unfolding.

Supplementary Table 7

Case	Force (pN)	Nb trajectories	Step at this force + correction (nm)	Corresponding contour length (nm)
N term, with SS	350	1	9.4	**10.1**
C term, with SS	250	11	6.6	7.2
	310	3	6.7	7.2
	350	5	7.2	7.7
			average	**7.3**
N term, no SS	250	2	15.9	17.6
	310	2	16.5	18.0
	350	3	16.8	18.3
			average	**18.0**

Supplementary Table 7. Estimation of released contour lengths in each unfolding scenario from MD trajectories of azurin.

Supplementary notes

Biochemical characterization of the samples:
a) *Ellman's test:*

The results of the Ellman's test for the three polyproteins (I27-Azu)$_4$, (I27-Azu$_{C26A}$)$_4$ and (I27-Azu$_{C112A}$)$_4$ is summarized below:

	Measured free thiols	Free thiols if SS bond is present in azurin	Free thiols if reduced cysteines in azurin
(I27-Azu)$_4$	14.7	14	22
(I27-Azu$_{C26A}$)$_4$	16.8	18	18
(I27-Azu$_{C112A}$)$_4$	11.6	10	18

The fact that the results obtained by the Ellman's assay (red) clearly follow the trend of the expected number of free cysteines present in the sample, and in particular that the wt protein (with the expected disulfide bond) exhibits a lower value than the C26 mutant (where the disulfide bond is not present) strongly suggests that the disulfide bond is preserved in the wt-azurin construct.

b) *ICP-OES experiments:*
Measurement of the copper content in the analysed protein samples provides quantification of the % of copper uptake for each sample (assuming that 100% of the proteins are correctly folded).

(I27-Azu)$_4$: 38.58%
(I27-Azu$_{C112A}$)$_4$: 11.38 %
(I27-Plastocyanin-wt)$_4$: 55.94%

The copper uptake for each of these proteins is, as expected, lower than 100%. This implies that some of the proteins within the polyprotein context did not bind the copper cofactor. This is likely to explain the two-state trajectories, lacking the mechanical intermediate, which we observed in the single molecule mechanical experiments. Crucially, the C112A mutation, while showing a lower percentage of copper uptake, does not completely abolish copper binding. These results are in line with our experimental observations showing that the C112A mutant exhibited a larger proportion of two-state trajectories. Quantitative correlation between the % of copper uptake and the % of trajectories showing a mechanical intermediate is difficult, since the copper uptake fluctuates within different individual purifications up to ±25%.

Supplementary Methods

Single Molecule Force-clamp experiments:

When working under force-clamp conditions, individual polyproteins were fished by pushing the cantilever onto the surface exerting a contact force of 500-1500 pN so as to promote the non-specific adhesion of the proteins on the cantilever surface. The piezoelectric actuator was then retracted to produce a set deflection (force), which was set constant throughout the experiment (~15-30 seconds) thanks to an external, active feedback mechanism while the extension was recorded. The force feedback was based on a proportional, integral and differential amplifier (PID) whose output was fed to the piezoelectric positioner. The feedback response is limited to ~3-5 ms. Thanks to the high-resolution piezoelectric actuator, our measurements of protein length have a peak-to-peak resolution of ~0.5 nm. Data of the force traces was filtered using a pole Bessel filter at 1 kHz.

Circular Dichroism experiments:

Experiments were conducted in the facilities of King's College London according to the following procedure:

UV & CD spectra of the 4 polyproteins in phosphate buffer were acquired on the Applied Photophysics Chirascan Plus spectrometer (Leatherhead, UK). 10mm and 0.5mm Quartz Suprasil rectangular cells (Hellma UK Ltd) were employed in the region 400-190nm. The instrument was flushed continuously with pure evaporated nitrogen throughout the experiment. The following parameters were employed: 2nm spectral bandwidth, 1nm stepsize and 1.5s instrument time per point. UV & CD spectra were buffer baseline corrected and measured at 23ºC. The far-UV CD spectra were smoothed with a window factor of 4 using the Savitzky-Golay method for better presentation. The far-UV CD spectra of the 4 polyproteins were then corrected for concentration and path-length and expressed in terms of $\Delta\varepsilon$ (M^{-1} cm^{-1}) per amino acid residue (MWt =113). Protein secondary structure contents were assessed using the Principle Component Regression method based on 16 known protein structures.

Ellmann's Reagent experiments (Determination of free protein sulfhydryls):

Protein samples were dissolved in 0.1M phosphate buffer, pH 7.4, containing 4M guanidine hydrochloride and adjusted to a final volume of 950 µl. A reagent blank (without sample) was prepared in a similar manner. After the addition of 50 µL of 20 mM 5,5'-dithiobis(2-nitrobenzoic acid) (DTNB) the sample was mixed, incubated at RT for 15 minutes and the absorbance at 412 nm was recorded. The amount of sulfhydryl was calculated using the extinction coefficient of DTNB in the presence of GuHCl (13,700 M^{-1} cm^{-1}). The number of free cysteines was determined using the protein and sulfhydryl molar concentration from each sample.

ICP-OES (Determination of copper content in the protein samples).

Experiments were conducted in the facilities of the University of Barcelona and the University of Edinburgh according to the following procedure:

The content of each sample eppendorf tube was totally digested by using a 1mL HNO_3 and 0.5mL H_2O_2 in a closed, Teflon reactor overnight at 90ºC.

Samples were then cooled down to RT and 15 ml of ultrapure water were added to each individual eppendorf tube containing the protein samples. The Cu content of the resulting solution was performed using an ICP-OES instrument (Perkin Elmer, Optima 8300) at working wavelengths of 324.752 nm and 327.393 nm. Calibration was performed using 5 standard solutions prepared by dilution of a 1000 ppm certified standard solution.

MD simulations

- Generalities

Constant-force steered molecular dynamics (MD) were performed on azurin starting from the PDB structure 4AZU solvated in a neutralized water box large enough in the direction of pulling to accommodate the partially unfolded protein. Simulations were performed both on the native, WT protein and on an oxidised form lacking the disulfide bond between Cys3 and Cys26. In the former case, the box size was approximately 6*6*18 nm and contained 61,482 atoms. In the latter case, the ~ 6*6*21-nm box contained 71,879 atoms. The NAMD 2.10 software was used to run the simulations on pure CPU or CPU/GPU nodes. Atomic parameters correspond to those in the CHARM36 force-field[1], while atomic charges for copper (II) and atoms of the 5 ligands were taken from quantum calculations[2]. We also constrained the organometallic bond distances following the procedure described elsewhere[2].

- Simulation procedure

The atomic positions were first minimized for 2,000 steps using the steepest-descent algorithm of NAMD. All subsequent simulations were run in the NPT ensemble, using the Langevin thermostat (0.2/ps) and the Langevin barostat of NAMD. Long-range electrostatics were treated using particle-mesh Ewald with a grid size of 1.2 $Å^{-1}$. Simulation timestep was 2 fs and bonds between hydrogen and heavy atoms were maintained rigid. After the minimization, the water was equilibrated around the protein, which was maintained fixed for 1 ns. All protein atoms except terminal C_α's were then allowed to move and equilibrated for 1 more nanosecond.

Starting from this previously equilibrated protein whose C-N axis was aligned along the longer edge of the box (direction of pulling), the C_α atom of the first residue was fixed while a constant force (250, 310 or 350 pN) was applied to the C_α atom of the last residue. In half of the trajectories, C_α from residue #1 was fixed and C_α from residue #128 was pulled; for the other half, C_α of residue #128 was fixed and C_α from residue #1 was pulled in the opposite direction. In the case of azurin, 20 such trajectories, lasting between 6 and 20 ns (until full possible extension was reached), were generated for the protein containing the disulfide bond (10 in each direction) in a ~18-nm long box; 9 trajectories total were generated in the absence of disulfide bond, in a slightly larger box (~21 nm). Simulation results are presented in Supplementary Tables 3-7.

- *Description of the unfolding dynamics in azurin*

MD simulations clearly revealed that, for both transition states, the rupture of the hydrogen bonds (HBs) between the involved β-sheets occurred through an unzipping mechanism because the force is applied parallel to the HBs

axes (Fig. 4A). In contrast to a protein containing such HBs perpendicular to the force, HB rupture occurs here sequentially, which explains why a relatively low force is sufficient to partially unfold the protein from one side. In the C-terminal unfolding scenario, the unfolding involves the rupture of the β7-β8 and β2b-β8 hydrogen-bonds, which occur at the same time; when the protein unfolds on the N-terminal side, it unzips by rupturing the hydrogen-bonds between β2b-β8, then β3-β6 and β1-β3 partially, before the remaining hydrogen-bonds of β1-β3 break. Interestingly, after one of these partial unfolding events, the protein flips and now exhibits β-sheets HBs perpendicular to the force on the side that is still folded (snapshots in Fig. 4C). Crucially, the key HBs whose rupture would lead to unfolding are now maintained perpendicular to the force and can resist to it in a cooperative manner. This suggests an explanation to the small occurrence (10% of the cases) of consecutive 6- and 9-nm steps. Indeed the low mechanical resistance of the organometallic bonds (Cu-Cys112 and Cu-His46, ~45 pN) makes them more likely to break before the HBs between β-sheets as long as they can cooperatively resist to the force.

The unfolding of the protein lacking its disulfide bond occurs preferentially from the N-terminal side. Interestingly, the time-course of β-sheets rupture events leading to unfolding slightly differs from that of the regular WT protein (detailed above). In this case, the protein generally unfolds as follows: first the hydrogen-bonds between β1-β3 break, followed by β1-β2a, β2b-β8, and finally β3-β6. This suggests that the presence of the SS bond locally rigidifies the protein and prevents the rupture of β1-β3, which clearly initiates the unzipping of the all the other β-sheets when the disulfide bond is not present. Instead, the main unfolding event in wt-Azu is the rupture of β3-β6 (which gives rise in the simulations to the presence of a short lived conformation around L=10 nm; Figure 4 in the main text).

- *MD simulations protocol of plastocyanin (holo- and apo- forms)*

Simulations of plastocyanin (PDB ID 3BQV) and apo-plastocyanin (#2PCY) were performed as previously described for azurin. As the active site of plastocyanin is very similar to that of azurin, with the exception of the fifth weak interaction with Gly45 of azurin being replaced by Pro38 in plastocyanin, we employed the same atomic charges as previously determined for azurin for His39, Cys89, His92 and Met97, and we took the charges on the carbonyl group and the C_α of Gly45 for the corresponding atoms of Pro38. This approximation is not expected to have any impact on the mechanical stability of the β-sheets located on the other side of the protein. As ligand-metal bonds display slightly different lengths than in azurin, we employed the equilibrium distances of the PDB structure to constrain these bonds during the simulations, with the exception of Pro38-Cu bond, which, since it is very long in the PDB (4.32 Å), we did not constrain as the interaction with the metal is probably extremely weak.

The rest of the simulation protocol was identical to that of azurin; solvation in a ~ 6*6*18 nm box, minimization and equilibration, and then pulling at a constant force in the range of 250-310 pN by fixing the C_α from the first residue and pulling on the C_α at the other end of the protein in the z-direction, and vice versa. 10 such trajectories (5/5) for plastocyanin and 10 for apo-plastocyanin were generated following this protocol, lasting a total of 5 or 10 ns each.

- *Effect of the organometallic bonds*

In classical MD simulations, organometallic bonds are challenging to describe as they usually have a covalent character that is very sensitive to the local geometry and to the environment. In usual forcefields, an interaction between 2 atoms is either described by non-bonded interactions (electrostatic and vdW forces) or by a bonded term, usually as an harmonic potential if they form a covalent bond. However, using electrostatic and van der Waals potentials to describe the interactions of the metal with its environment (and in particular with the ligands) would cause the metal to leave the active site as it would be better solvated in water: we have checked that this happens very quickly in the simulations in the case of azurin. Therefore, additional harmonic potentials are necessary to give some covalent character to these bonds and thus to keep the geometry of the active site correct, the main drawback being that they cannot be broken during the simulation, nor changed in energy due to local changes of the environment or the ligand-field geometry.

For the purpose of the current work, such an approximation is very reasonable, for 2 main reasons. First, the mechanical unfolding events studied here all start with the breaking of key sets of hydrogen-bonds that are located on β-sheets placed on the other side of the protein, next to both termini. The breaking of metallic bonds is only involved at the very end of the unfolding events, when one portion of the protein is extended. This bond-breaking is not possible in the simulations but can be artificially accounted for when comparing the simulation observed extensions to that of the experiments (see Simulation section of azurin).

Second, we have simulated plastocyanin in its regular, metal-bonded form, but also in its apo- structure, lacking the metal. As seen in Supplementary Tables 5 and 6, in both cases we observe a very similar ratio of N-terminal to C-terminal unfolding events (around 80/20%, respectively, Supplementary Fig. 13), suggesting that the metal active site has actually none or very little influence on the unfolding mechanism of plastocyanin. Moreover, the first step of the mechanical unfolding in both scenarios is identical in each case (rupture of β1-β3 for N-terminal unfolding; rupture of β2b-β8 and β7-β8 for C-terminal unfolding). The main role of the metal in the current investigation is thus to reveal the intermediate states of the protein unfolding that would normally totally unfold in the absence of metal.

- *Predictions of released contour lengths from MD trajectories*

MD simulations can be used to predict the increase in contour lengths of unfolding in the two scenarios (N-terminal and C-terminal unfolding). To this end, we have followed the following procedure (Supplementary table 7): we first measured, for each simulation, the extension of the protein during unfolding, i.e., the difference between the final and original end-to-end distances projected onto the z-axis. Then, since in the simulations unfolding does not occur up to Cys112 for C-terminal unfolding (rather, up to Met121) and up to His46 for N-terminal unfolding (rather up to Gly45), we artificially added the extra-lengths corresponding to the release of the amino-acids in between (Cys112-Met121 and Gly45-His46), at each simulation force, using the worm-like chain model with a

contour length per residue of 0.38 nm and a persistence length of 0.4 nm. This correction is very moderate for N-terminal unfolding but more substantial for C-terminal (about 2 nm), and it is force-dependent. By doing so, we obtained the average extension for each unfolding scenario and at each probed force. The last step is to convert these extensions at finite forces into that measured in the experiments, which correspond to the contour lengths obtained by applying a WLC model fit of the data. This conversion is done once again using the WLC model on the «free» amino-acids, i.e. those that are extended and that do not belong to the folded structure anymore in each intermediate (between each free-termini and the active site, minus the portion between the disulfide bond, if applicable). Following this procedure, we obtained the following values: 7.3 nm (N-terminal, with SS bond present); 10.1 nm (C-terminal, with SS bond present); 18.0 nm (C terminal, without SS bond). The agreement with the experimental measurements (respectively 6, 9 and 19 nm) and the schematic predictions (Supplementary Figs. 1 and 8) is very good.

Supplementary References

1. Huang, J. & MacKerell, A.D., Jr. CHARMM36 all-atom additive protein force field: validation based on comparison to NMR data. *Journal of computational chemistry* **34**, 2135-2145 (2013).
2. van den Bosch, M. *et al.* Calculation of the redox potential of the protein azurin and some mutants. *Chembiochem* **6**, 738-746 (2005).
